# The Mechanisms of Lead Toxicity in Living Organisms

**DOI:** 10.3390/jox15050146

**Published:** 2025-09-11

**Authors:** Anastasiia Generalova, Slavena Davidova, Galina Satchanska

**Affiliations:** 1 UPIZ Educational and Research Laboratory of Biology-MF, New Bulgarian University, Montevideo Blvd. 21, 1618 Sofia, Bulgaria; f116172@students.nbu.bg (A.G.); stdavidova@nbu.bg (S.D.); 2 Department of Natural Sciences, New Bulgarian University, Montevideo Blvd. 21, 1618 Sofia, Bulgaria

**Keywords:** Pb pollution, Pb toxicity, bioaccumulation, neurotoxicity, oxidative stress, ion mimicry, environmental remediation, molecular mechanisms

## Abstract

Lead (Pb) is a non-essential, toxic heavy metal with no known biological function that has caused widespread environmental contamination throughout human history. Pb toxicity represents one of the most persistent environmental health challenges, with no safe exposure threshold identified. The metal demonstrates remarkable persistence in biological systems, with approximately 90% of it stored in bone tissue for decades, mimicking calcium due to its similar ionic properties. Contemporary contamination primarily stems from mining activities, battery manufacturing, electronic waste recycling, and deteriorating infrastructure. Pb enters organisms through multiple pathways and causes severe health impacts across all biological systems, with particularly devastating neurodevelopmental and bone effects in children and cardiovascular and reproductive consequences in adults. On a molecular level, Pb disrupts cellular processes through ion mimicry, replacing essential metals in enzymes and proteins and leading to mitochondrial dysfunction, oxidative stress, DNA damage, and epigenetic modifications. This review examines the sources of Pb pollution and its toxicological impacts on bacteria, fungi, plants, animals, and humans. It explores the molecular mechanisms underlying these effects, including neuroinflammation, genotoxicity, and cell death pathways. The paper considers current approaches for Pb removal from contaminated environments and therapeutic interventions for Pb poisoning.

## 1. Introduction

The name “lead” originates from the Anglo-Saxon word of unknown origin, while its chemical symbol, Pb, is derived from the Latin word plumbum [1]. Historical records indicate that lead was widely used in ancient societies for pipes, cookware, cosmetics, and even wine storage, which significantly contributed to environmental contamination [2]. In Table 1, some of Pb’s physical and chemical properties are described.

Although the use of leaded gasoline has been banned in most countries since the 20th century, today, the primary sources of lead exposure include mining, battery manufacturing, smelting, electronic waste recycling, and the deterioration of lead-based paints, as well as aging plumbing systems that continue to contaminate drinking water [2,3]. This topic is extremely relevant as Pb is characterized by its persistence and continuous release into the blood from bones, which permanently poisons all tissues, organs, and cellular functions. Pb easily penetrates cells because it mimics Ca, an essential element for all living organisms. Pb is widespread in polluting the environment—including soil, water, air, and food—leading to easy transfer of Pb into humans and all living organisms. This review aims to summarize information and address the issue of the source of many current illnesses and their increasing prevalence. It also explores the probable role of Pb in the emergence of new diseases and the decreased age of people who have cancer, such as 30- to 40-year-olds.

### Systematic Methodology

This study is based on a large-scale landscape analysis of Pb research across scientific databases, including Scopus, Web of Science Core Collection, PubMed, ScienceDirect, MDPI, Google Scholar, and others. We initially collected approximately 950 relevant peer-reviewed articles. After selection, we reduced them to 400, and now to 166. The targeted keywords used were “lead toxicity mechanisms”, “lead contamination sources”, “Pb bioaccumulation”, “Pb molecular mechanisms”, “oxidative stress and lead”, “ion mimicry Pb”, “lead in soil, water, and air”, and “Pb exposure health effects”.

The search included international and regional studies from Europe, China, India, Africa, the Middle East, and North and South America, with the aim of comparing Pb concentrations in environmental media and living organisms to allowable standards set by the WHO, the FAO, the European Union, the US EPA, China’s GB standards, and other national screening levels.

The initial search was limited to publications from a 10-year period (2015–2024), with a focus on the most recent data (2020–2024) to ensure up-to-date coverage of toxicological findings and remediation approaches. The primary selection criteria were as follows:

1. Studies dealing exclusively or predominantly with lead (Pb) as the contaminant.
2. Research reporting quantitative Pb concentrations in soil, water, air, plants, animal tissues, or human samples.
3. Articles describing toxicological effects (neurotoxicity, oxidative stress, mitochondrial dysfunction, reproductive toxicity, etc.) or molecular mechanisms of Pb action in living organisms.
4. Studies providing comparisons to international or national permissible limits (e.g., 0.01 mg/kg Pb in food, 5 μg/L in drinking water, 0.5 μg/m^3^ in air, or 50–300 mg/kg in soil).

The secondary selection involved reviewing abstracts and full texts to assess methodological quality, data reliability, and relevance to the research objectives. Preference was given to experimental studies, systematic reviews, and meta-analyses with clearly described sampling methods and statistical analyses.

**Figure 1:**
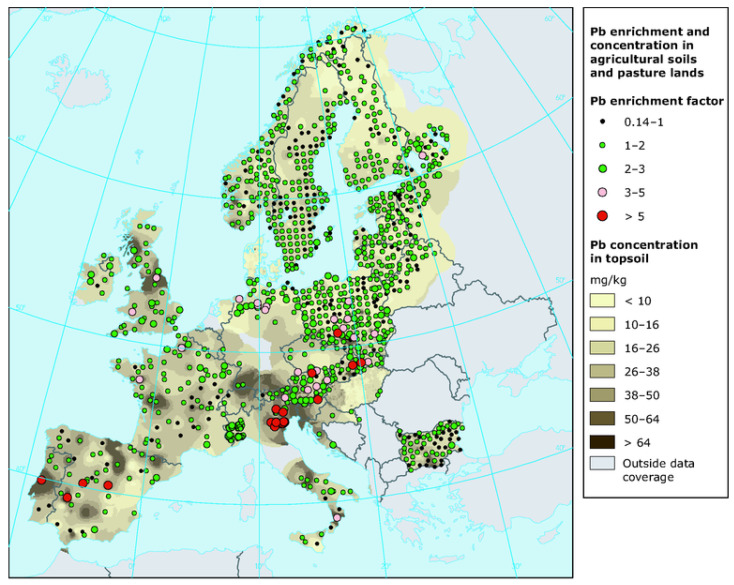
Concentration of Pb in topsoils in Europe (0–25 cm) [5]. This figure is taken from the European Environment Agency (https://www.eea.europa.eu/en) (accessed on 7 July 2025).

The WHO classifies Pb as one of the ten most hazardous chemical elements for humans. In 2016, data from the Institute for Health Metrics and Evaluation (IHME) indicated that Pb poisoning resulted in 540,000 deaths globally. Most of these fatalities occurred in low-income countries. Pb contributes to 64% of primary idiopathic intellectual disabilities, 3% of ischemic heart disease cases, and approximately 3.1% of heart attacks worldwide [4]. Pb has long been a part of human life; for centuries, its compounds were used to sweeten sour wines, and it is still produced today in Pb-Zn processing plants, as well as in paint factories, photocopiers, and automotive and jet fuels to boost octane levels. According to the WHO, Pb emissions into the atmosphere come from various recycling activities. Pb is also utilized in the manufacturing of batteries, rivets, ammunition, ceramic glazes, jewelry, toys, and traditional medicine. A significant portion of Pb is used in car batteries [4]. The International Lead Association (ILA) reports that global annual primary Pb production is around 5 million tons, valued at approximately $15 billion, which has doubled over the past decade. When released into outdoor air, Pb becomes part of street dust, potentially causing allergic reactions in the population. The US Environmental Protection Agency (EPA) recommends that Pb levels in city air should not exceed 9 µg/m^3^. In the USA, Pb is one of six required air quality parameters, alongside CO, NO_2_, O_3_, SO_2_, and PM2.5 and PM10 particulate matter. In freshwater, Pb concentrations typically range from 1 to 10 µg/L [4]. Figure 1 depicts the concentration of Pb in agricultural soils and pasture lands in Europe.

Lead is a non-essential, highly toxic metal with no known biological function. Once absorbed—mainly through inhalation or ingestion—it is distributed to the brain, liver, kidneys, and bones. Lead mimics essential metals such as calcium and zinc, disrupting cellular signaling and enzyme activity. It induces oxidative stress, inflammation, and interferes with gene expression and DNA repair mechanisms [2,6].

Due to its long biological half-life and poor excretion, lead bioaccumulates in soft and hard tissues over time. Chronic exposure affects multiple organ systems, particularly the nervous, renal, cardiovascular, hematopoietic, and reproductive systems. Children are particularly vulnerable due to their higher absorption rates and ongoing neurodevelopment. Even low blood lead levels are associated with cognitive and behavioral impairments [2,6]. According to the Global Burden of Disease Study, lead exposure contributed to approximately 900.000 deaths globally in 2019, reinforcing its classification as a probable human carcinogen [2]. Pb and inorganic compounds are classified in Group 2A of probable carcinogens according to the IARC (International Agency for Research on Cancer—https://monographs.iarc.who.int/list-of-classifications, accessed on 9 July 2025), while organic compounds are classified in Group 3.

Classification of Pb by the WHO as one of the most hazardous heavy metals necessitates the summarization of Pb pollution sources.

## 2. The Origin of Pb Pollution

**Figure 2:**
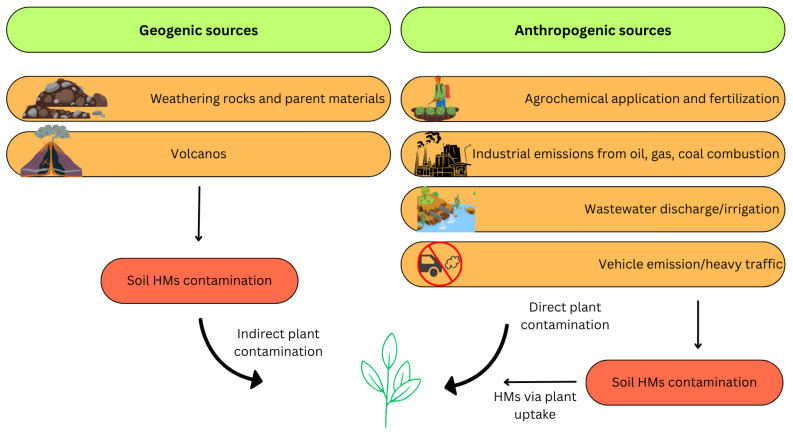
Principal origins of lead contamination in the soil–plant system [9]. In the soil–plant system, heavy metal contamination of plants occurs through two principal pathways: indirect and direct. In the indirect pathway, HMs from geogenic sources (e.g., weathering of rocks, volcanic activity) and anthropogenic sources (e.g., agrochemical application, industrial emissions, wastewater irrigation, vehicle exhaust) accumulate in the soil, where they dissolve into the soil solution and are subsequently absorbed by plant roots. These metals are then translocated via the vascular system to edible plant parts, posing potential health risks for both plants and humans. The direct pathway involves the deposition of HM-containing particulates directly onto aerial plant surfaces through atmospheric fallout from industrial processes, vehicular traffic, or resuspended contaminated dust. In this case, metals may penetrate leaf tissues through stomata or remain on the surface. The soil HMs pathway serves as a central component in both processes, acting as a long-term reservoir for root uptake in the indirect pathway and as a source of contaminated dust contributing to direct surface deposition. A study conducted in India [10] on agricultural soils producing wheat, rice, maize, leafy vegetables, and mustard seeds confirmed that multiple anthropogenic activities, including emissions from thermal power plants, cement factories, and intensive agrochemical use, contributed to HM contamination through both direct and indirect routes.

Lead is a toxic heavy metal that has been extensively utilized throughout human history, resulting in its widespread environmental distribution. Despite natural sources, such as volcanic emissions and erosion, most of the environmental Pb burden is of anthropogenic origin (Figure 2) [2]. The industrial use of lead peaked during the 20th century with its incorporation in gasoline additives (e.g., tetraethyl lead), batteries, paints, ceramics, and plumbing systems [7]. Even after regulatory efforts to eliminate leaded gasoline, Pb emissions from smelting, battery recycling, and mining continue to be prominent pollution sources worldwide [8].

Historically, the use of lead in urban infrastructure—such as water pipes, roofing, and household paint—resulted in significant accumulation in soil and dust, especially in densely populated or industrialized regions. Additionally, in the Pb crystal industry, Pb glass carving also causes professional diseases among workers. In the United States, legacy contamination from such uses has led to persistent soil Pb levels exceeding standard thresholds, particularly in urban neighborhoods with older housing stock [11]. Likewise, regions in Poland, such as Upper Silesia, experienced prolonged air pollution due to nearby heavy industry, which contributed to substantial lead deposition in forest and park soils [12].

In many low- and middle-income countries, lead exposure remains an urgent health concern. Informal recycling of lead-acid batteries, illegal smelting, and adulteration of consumer products (e.g., spices, cosmetics) are major contributors to environmental and human contamination [8,13]. Sustained environmental exposure, even at low levels, persists due to Pb’s long environmental half-life and strong binding to soil and sediment matrices [14].

The persistence of lead in soil is influenced by factors such as pH, organic matter content, and soil texture. For instance, acidic soils or those rich in organic carbon tend to enhance Pb mobility and bioavailability, posing risks to soil microflora and indirectly affecting plant health and nutrient cycling [12]. In Polish mountain landscapes affected by industrial emissions, these soil conditions amplified Pb toxicity and microbial stress, particularly in the upper soil layers [12].

Lead contamination of water systems is another concern, primarily through runoff from polluted soil or the corrosion of plumbing components containing lead. Even minimal levels of Pb in drinking water or food can cumulatively harm biological systems. This is due to Pb’s poor excretion and high affinity for biological molecules [2,14]. These properties make Pb a persistent threat to environmental and public health, particularly in developing regions where monitoring and remediation infrastructure is lacking.

### 2.1. Natural Occurrence of Pb

#### 2.1.1. Soil

Pb naturally occurs in trace concentrations within the Earth’s crust, constituting approximately 0.002% of its composition [15]. Background concentrations of lead in soil primarily arise from the weathering of bedrock containing lead-bearing minerals and other geogenic processes, such as sea spray and volcanic activity, particularly in regions with significant natural mineral deposits [15,16].

Over time, soils may become enriched with lead through the slow breakdown of parent materials such as galena (PbS), anglesite (PbSO_4_), and cerussite (PbCO_3_). These minerals release lead ions into the soil matrix during prolonged physical and chemical weathering processes [17]. The mobility and chemical behavior of naturally sourced lead in soil are strongly influenced by local biogeochemical conditions, particularly soil pH and redox potential. In acidic soils (pH < 4.0), lead is more likely to exist in a soluble ionic form (Pb^2+^), increasing its bioavailability and potential for plant uptake [17]. Conversely, in alkaline conditions (pH > 7.0), lead tends to precipitate as insoluble compounds such as Pb(OH)_2_ or PbCO_3_, reducing both its mobility and toxicity [17]. In areas rich in iron (Fe) and manganese (Mn) oxides, naturally occurring lead is often adsorbed onto these mineral surfaces. In this way, Pb movement and uptake by plants are limited [16].

Additionally, lead released through natural weathering may form long-lived complexes with clay minerals and organic matter, further influencing its persistence and behavior in the soil environment [16]. High-molecular-weight organic compounds can bind Pb ions, forming stable complexes that reduce lead solubility and limit biological interactions. However, low-molecular-weight organics may increase lead mobility by forming soluble chelates [17].

Microbial activity is another critical factor affecting the fate of naturally present lead. Soil microbes can influence pH and redox conditions through metabolic processes, thereby altering lead speciation and its ecological risk profile [17]. Some bacteria, particularly under anaerobic conditions, can convert lead into less mobile sulfide forms (e.g., PbS), effectively immobilizing the metal [17]. Extensive research on the influence of Pb and heavy metals on soil bacteria was conducted by Satchanska et al. [18,19,20,21]. Undoubtedly, the results obtained from the long-lasting experiments showed a shift in microbial community structure in polluted environments. Pilot biotechnologies with entrapped in polyethylene oxide cryogels, environmental heavy-metal tolerant bacteria, which we developed by our team [22,23,24,25,26,27,28,29,30].

#### 2.1.2. Water

Based on the analyzed studies, lead (Pb) concentrations in marine and freshwater environments show significant variation and contamination patterns. In marine sediments from Mallorca’s southwest coast, background Pb levels averaged 10.1 mg/kg. Still, concentrations near wastewater discharge points reached heavily polluted levels, with three samples at Baluard station exceeding probable effect levels (PEL) according to National Oceanic and Atmospheric Administration (NOAA) standards. In contrast, six additional samples fell between the threshold and PEL [31]. The Red Sea coastal sediments showed elevated contamination with mean Pb concentrations of 57.8 mg/kg in Egypt, 39.7 mg/kg in Saudi Arabia, and 96.67 mg/kg in Jordan (Table 2)—all significantly above the upper continental crust reference level of 17.0 mg/kg. One extreme case in Egypt’s Hurghada region reached 865 mg/kg, indicating serious non-carcinogenic health risks [32].

Freshwater systems demonstrate comparatively lower but still concerning levels of Pb. In China’s Fenghe River Basin, water Pb concentrations ranged from 0.70 to 1.51 μg/L, remaining below the WHO standard (10 μg/L). In contrast, sediment concentrations averaged 30.2 mg/kg, exceeding the regional background value of 21.4 mg/kg at all sampling points [34]. African water bodies show alarming contamination levels, with WHO drinking water standards (0.01 mg/L) exceeded by ratios of 82–669 times in various locations. Surprisingly, Lake Victoria is also included in the list, highlighting the urgent need for improved water treatment and monitoring systems across different aquatic environments [35].

Lead contamination in water arises primarily from anthropogenic sources, including industrial discharge, urban runoff, aging infrastructure, and untreated domestic wastewater [3]. In urban environments, rain and snowmelt mobilize historical lead deposits from roads, rooftops, and construction surfaces, allowing them to enter surface water bodies. For example, according to the EPA in the United States, it has been estimated that approximately 3000 tons of lead are mobilized each year by storm runoff from residential areas of cities with more than 100,000 inhabitants [3]. Although lead concentrations in the water column may diminish through dilution or sedimentation, sediments located near urban and industrial areas retain significant amounts of lead. In this mode, it serves as a persistent secondary source of contamination [3]. Furthermore, domestic waste from urban centers—especially where wastewater treatment is insufficient—introduces various heavy metals, including lead, into river systems [36]. In remote regions, such as the Arctic and subarctic, exposure remains a concern due to reliance on untreated water sources and older infrastructure, particularly among Indigenous populations [37].

Industrial processes constitute a major contributor to lead input in aquatic environments. Heavy industries such as smelting, coal processing, tanning, and chemical manufacturing discharge significant volumes of effluents, often containing lead and other toxic metals, into surface and groundwater systems [36]. When inadequately treated or released directly, these discharges severely compromise water quality and accumulate in sediments [36]. Alongside this, agriculture also acts as a widespread non-point source of heavy metal pollution. Fertilizers, pesticides, and other chemical inputs applied to farmlands can contain lead and related metals, which are then mobilized by rainwater runoff into nearby aquatic systems [36]. Continuous input from agricultural activities has led to notable long-term contamination in regions of intensive farming [36].

Improper waste management practices also play a significant role in lead contamination. Landfills and open dumping sites lacking containment systems allow leachates enriched with heavy metals, including lead, to infiltrate surrounding soils and water bodies [36]. Municipal solid waste, which often contains a mix of industrial, household, and healthcare refuse, contributes to this problem when it is dumped without segregation [3,36]. Over time, toxic substances from decomposing waste migrate into surface and groundwater via percolation or runoff [3,36]. Additionally, natural processes, such as the weathering of rocks and minerals, can release trace amounts of lead into water systems; however, these sources are generally less significant compared to anthropogenic inputs [36].

#### 2.1.3. Air

Lead enters the atmosphere and binds to particulate matter that can be transported over long distances and subsequently deposited onto soil and dust surfaces, creating a significant exposure pathway for humans through both inhalation and ingestion [38]. Regulatory standards for workplace air exposure vary significantly, with the Occupational Safety and Health Administration (OSHA) establishing limits of 0.5 mg/m^3^ for elemental lead in general industry and 50 μg/m^3^ in construction environments. In contrast, the WHO maintains an air quality standard of 0.5 μg/m^3^, which is 10 to 100 times lower [38,39].

Atmospheric monitoring studies have documented varying lead concentrations across different urban and industrial environments. In the industrial areas of Coimbatore city, suspended particulate matter contained lead concentrations ranging from 5 to 7 μg/m^3^, with lead ranking fourth among the heavy metals detected (Fe > Zn > Cu > Pb > Ni > Cr) [40]. Dust fall particles in Zarand, Iran, showed lead concentrations of 1.01 mg/kg with an enrichment factor of 194.2, indicating predominantly anthropogenic origins from vehicle and industrial emissions [33]. These atmospheric lead particulates pose significant health risks through inhalation, particularly affecting the cardiovascular and respiratory systems, and contribute to soil and household dust contamination [38,40]. Table 3 summarizes the maximal permissible limits of Pb in food, drinking water, soil, and air.

### 2.2. Anthropogenic Pb Sources

Industrial and agricultural activities synergistically contribute to environmental lead contamination through multiple pathways. Rapid industrialization in developing nations has intensified lead release through manufacturing processes (such as battery production, metal smelting, ammunition, ceramic glazes, jewelry, toys, etc.), waste incineration, and improper disposal of effluents. At the same time, agricultural practices involving contaminated fertilizers, pesticides, and wastewater irrigation perpetuate soil contamination [45].

Atmospheric deposition represents the predominant contributor to agricultural lead contamination, particularly in industrialized regions. In China, atmospheric deposition accounted for 80–94% of total lead inputs to agrarian soils between 2006 and 2015, compared to 43–85% between 1999 and 2005. Coal combustion contributed 22% and vehicle emissions 78% to lead in atmospheric dust in Northeast China [46]. Industrial activities, including fossil fuel combustion, manufacturing processes, and mining operations, constitute primary anthropogenic sources. Measurements of atmospheric deposition in China were conducted at 72–178 monitoring sites across various regions, primarily located in agricultural lands and farms, and intentionally placed away from industrial or mining sources to reflect average national conditions. Overall, the data indicate a 5% increase in atmospheric deposition lead inputs, which can be attributed to the expansion of farmland area by approximately 10.6% [46]. Phosphate fertilizers contain average lead concentrations of 2.9 mg/kg across 12 European countries [46].

Metal cookware emerges as a significant and underrecognized source of lead exposure due to manufacturing practices that utilize contaminated scrap materials. Aluminum and brass cookware frequently exceed 100 ppm lead content (0.01%), with some brass components reaching 10,000 ppm (1%), particularly in handles [47]. Traditional Afghan pressure cookers have been found to contain lead levels of up to 69,000 ppm in the pressure relief vent pipes. At the same time, Hindalium cookware leached lead amounts exceeding childhood dietary limits by 1.400-fold after 24 h of contact with acidic solutions [47].

Occupational exposure is the most prevalent anthropogenic source of lead in the Middle Eastern and North African regions, accounting for 26.5% of documented cases [13]. Workers in petrol stations, battery manufacturing, smelting operations, and automotive repair face elevated exposure risks, with secondary contamination affecting families through contaminated clothing [13]. Cosmetic products, particularly traditional kohl, represent the second most common source, accounting for 23.5% of cases. In contrast, residential factors, including lead-based paint in buildings older than 20 years, account for 20.6% of exposure sources [13].

### 2.3. Global Pb Pollution by Continents

**Figure 3:**
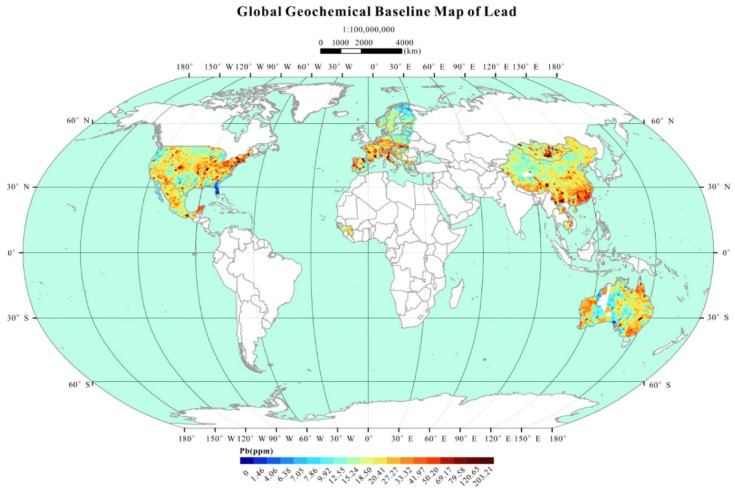
Global map showing lead coverage, which accounts for 27% of Earth’s land surface [48].

Lead contamination is a global issue and affects all continents. Figure 3 below illustrates a global map of lead pollution, which affects 27% of the Earth’s surface.

#### 2.3.1. Pb Pollution in Africa

Lead exposure continues to be a significant issue in South Africa. A study by Schirnding et al. [49] investigated blood lead levels in young children from Aggeneys, a lead mining town in South Africa’s Northern Cape Province, and compared them to children in Pella, a community about 40 km away. The findings indicated that children in Aggeneys had an average blood lead level of around 16 μg/dL, while those in Pella averaged 13 μg/dL. Overall, children with higher blood lead levels tended to perform worse at school than their peers [49].

In a study by Naicker et al. [50], the aim was to explore the connection between blood lead levels and socio-behavioral issues in young adolescents. Beginning in 1989, this long-term prospective study tracks the health and well-being of children born in the Greater Johannesburg area. The total analytical sample included 1.041 adolescents (487 males and 554 females). The geometric mean blood lead level was significantly higher in boys (6.0 μg/dL) compared to girls (4.5 μg/dL), with a *p* value of less than 0.001. Elevated blood lead levels are linked to anti-social and destructive behaviors among boys in early adolescence [50].

A 2021 study by Fisher et al. [51] collected water samples from 261 community water systems, including handpumps and public taps, in rural Ghana, Mali, and Niger. Lead was most often found at concerning levels in components and samples. Lead mass fractions surpassed the International Plumbing Code (IPC) recommended limit of 0.25% wt/wt in 82% (107/130) of systems tested. Brass components were particularly problematic, with 72% (26/36) exceeding IPC standards. The presence of a brass component increased the expected lead concentration in drinking water by 3.8 times. Overall, lead levels exceeded World Health Organization (WHO) guideline values in 9% (24/261) of samples across the countries [51].

#### 2.3.2. Pb Pollution in Asia

A study in 2007 by Pusapukdepob et al. [52] assessed environmental lead exposure and its link to blood and teeth lead levels and IQ among residents near lead mining sites in Kanchanaburi, Thailand. The study involved 215 villagers from six villages. Participants completed IQ tests based on Raven’s Standard Progressive Matrices. Soil, blood, and teeth samples were collected and analyzed for lead. Results showed that soil, vegetables (including mint, bitter gourd, Chinese watercress, basil, and turmeric), and meat (such as fish and shellfish) had lead levels above the standards. Exposed individuals had blood and tooth lead levels over 10 μg/dl and 10 μg/g, with an average IQ of 82.70 (*p* < 0.05). Non-exposed had lower lead levels and an average IQ of 96.14 (*p* < 0.05). The health risk of low IQ was 5.6 times higher in exposed individuals (*p* < 0.05). IQ scores in the exposed group were inversely correlated with blood and tooth lead levels (r = 0.397 and 0.129, *p* < 0.05). The findings suggest children exposed to environmental lead accumulate it in their bodies, affecting cognitive development [52].

A 2015 study by Dai et al. [53] analyzed blood lead, serum uric acid, and hyperuricemia among 2.120 adults in a lead-polluted Chinese region. Blood lead correlated positively with serum uric acid in both men (r = 0.095, *p* = 0.001) and women (r = 0.134, *p* < 0.001). In men, blood lead, age, smoking, education, triglycerides, and serum creatinine are independently linked to uric acid. In women, blood lead, BMI, and triglycerides showed independent associations. Higher blood lead levels increased hyperuricemia risk in women (OR = 2.190; 95% CI: 1.106–4.338; *p* = 0.025), but not in men. Exposure affects serum uric acid in both sexes, especially women, and is linked to hyperuricemia in women only [53].

A large survey by Norton et al. [54] analyzed 1.578 rice grain samples from markets in 13 countries and fields in 6 countries for lead content. Among the market samples, only 0.6% exceeded the Chinese and EU limit of 0.2 μg g^−1^ lead (excluding samples from known contaminated or mine-affected areas). When comparing the samples to the FDA’s provisional total tolerable intake (PTTI) for children and pregnant women, only those consuming large amounts of rice risk exceeding the PTTI from rice alone. Japan had the lowest mean rice lead level at 0.004 μg g^−1^, while China showed the highest: 0.046 μg g^−1^ in regions of unknown contamination and 0.185 μg g^−1^ in regions affected by contamination or mining. Following China, Nepal, Ghana, India, and Sri Lanka had the highest average rice lead levels, with all other countries averaging below 0.015 μg g^−1^ [54].

#### 2.3.3. Pb Pollution in Europe

In 2002, Haider et al. [55] conducted two surveys of Pb in Vienna’s drinking water, involving 288 sites and 1768 samples. The first, with 51 sites, used a field experimental approach to examine the effects of time of day, floor level, and flushing, focusing on sites built before 1945, suspected of lead plumbing, representing the worst case. The second involved 237 randomly selected sites, stratified by consumption time, to assess the current situation. Each included one sample without flushing and three with 1, 3, and 10 L of flushing. Significant lead decreases were observed, with higher levels on upper floors indicating household plumbing as the main contamination source. Compared to other European countries, fewer samples exceeded the guideline levels (50 µg/L current standard, 10 µg/L target) [55].

The study by Peña-Fernández et al. [56] in 2014 examined lead exposure among children and adolescents living in Alcalá de Henares, Spain. The city was divided into four zones to analyze the effect of residence on Pb levels, considering variables such as age and gender. The research included 115 children aged 6–9 years and 96 adolescents aged 13–16 years. Results showed a significant difference in Pb levels in adolescents’ hair based on gender and residence area (*p* < 0.001 and *p* < 0.01). No significant differences were found in children’s hair Pb levels by gender or location. Pb levels were notably higher in children than adolescents (1.48 vs. 0.70 μg/g, *p* < 0.001), and adolescent females had higher levels than males (0.53 vs. 0.77 μg/g, *p* < 0.001). The correlation between residence area and Pb levels in adolescents likely reflects differences in diet and socioeconomic status [56].

#### 2.3.4. Pb Pollution in North America

An urban Philadelphia neighborhood with soil lead contamination was studied in 2017 by Bradham et al. [57] to assess the correlation between soil Pb (total and bioaccessible) and children’s blood lead levels (BLL). Soil samples from 38 homes showed total Pb from 58 to 2821 mg/kg and bioaccessible Pb from 47 to 2567 mg/kg, with children’s BLLs from 0.3 to 9.8 μg/dL. Hierarchical models revealed that total Pb explained 23% of BLL variability, while bioaccessible Pb explained 26%. Bootstrapping confirmed a significant model fit increase with bioaccessible Pb, with 99.0% of bootstraps positive. BLL increase estimates were 1.3 μg/dL per 1000 mg/kg total Pb and 1.5 μg/dL per 1000 mg/kg bioaccessible Pb. Age did not significantly impact BLL predictions [57].

Obeng-Gyasi et al. [58] assessed soil Pb exposure risk in Greensboro, North Carolina, using field sampling, statistical analysis, and machine learning. They collected 2.310 samples from 462 households’ dripline, yard, and street side, testing for Pb and integrating data on Pb sources, soil properties, and demographics. Results showed 43% of samples exceeded guidelines, with significant racial disparities—soil Pb at the dripline increased by 19% for every 25% rise in residents identifying as Black [58].

Leibler et al. [59] conducted a cross-sectional study in Greater Boston with 51 backyard chicken owners, analyzing 201 eggs and 48 soil samples for lead content using ICP-MS and XRF. The USEPA’s IEUBK model assessed the impact of backyard egg consumption on children’s blood lead levels (BLLs) across four scenarios. Lead was detected in 98% of eggs, with concentrations ranging from below detection to 1798 μg/g, showing a strong correlation between egg and soil lead levels (r = 0.64; *p* < 0.001). Frequent consumption of contaminated eggs could increase BLLs by 0.9–1.5 μg/dL in children under 7, while moderate exposure raises BLLs by 0.1 to 0.8 μg/dL. The study shows backyard eggs can contribute to childhood lead exposure, and soil remediation may reduce this risk [59].

#### 2.3.5. Pb Pollution in South America

In 2003, Espinoza et al. [60] measured blood lead levels in urban children (n = 2510) and women (n = 874) postpartum across districts of Lima and Callao, aiming to correlate these levels with exposures. The cross-sectional study, conducted from July 1998 to January 1999, employed three sampling methods within the government school system and public hospitals. Staff collected finger-stick blood samples following protocols to minimize contamination. Blood and environmental samples were analyzed on-site with portable voltameters. Multivariate regression assessed the impact of predictors on blood lead levels. Children’s average was 9.9 µg/dL, with 29% >10 and 9.4% >20 µg/dL. Women averaged 3.5 µg/dL, with 2.4% above 10 µg/dL. Significant location differences were noted, especially near the port of Callao. Children near mineral storage had a mean of 13 µg/dL, compared to 7.1 in others. Exposure to port sources increased levels by over 13 µg/dL. In Lima, higher vehicular traffic is associated with an increased risk of levels exceeding 10 [60].

In 2012, Anticona et al. [61] studied risk factors linked to elevated blood lead levels (BLLs) in children from two communities along the Corrientes River basin in the Peruvian Amazon. Children aged 0–17 were tested for BLLs, hemoglobin, and body measurements. Out of 208 children—88 from 23 households in Peruanito and 120 from 28 households in Santa Isabel—27.4% had BLLs over 10 µg/dL. The geometric mean BLL was 8.7 ± 4.0 µg/dL (range 3.0–26.8). Overall, linear regression showed age was positively associated with BLLs (*p* < 0.05). Logistic regression revealed that boys had 2.12 times higher odds of BLL > 10 µg/dL compared to girls (*p* < 0.05). Among children aged 0–3, those whose mothers had BLLs > 10 µg/dL had 45.0% higher odds of elevated BLLs than children whose mothers had BLLs below 10 µg/dL (*p* < 0.05). The main risk factors identified were older age, male gender, and maternal BLL > 10 µg/dL. The increased risk observed in boys aged 7–17 suggests that certain activities, such as fishing and hunting, may contribute to their exposure [61].

Machado et al. [62] investigated soil contamination near a metallurgical plant in Santo Amaro, Brazil, correlating lead and cadmium levels with past emissions and debris. Most soil samples exceeded safety limits, especially near the plant, with about 80% of homes showing lead above 300 ppm and nearly half exceeding 900 ppm, indicating the role of debris. Cadmium levels are less concerning, likely due to higher mobility. Atmospheric and backyard samples show similar patterns, though debris impact is less clear [62].

#### 2.3.6. Pb Pollution in Oceania

Research by Taylor et al. [63] examined how lead and SO_2_ emissions from the Nyrstar Port Pirie smelter in South Australia impact health and air quality standards. Analyzing data from SA Health and SAEPA, regression analysis from 2003 to 2017 revealed that rising SO_2_ levels are linked to increased respiratory visits, especially among children, with a significant association (*p* < 0.05). The study recommends that the annual geometric mean air lead stay below 0.11 μg/m^3^, and 24-month-olds’ air lead should not exceed 0.082 μg/m^3^, to keep children’s blood lead below 5 μg/dL [63].

Mount Isa, Queensland, is one of three Australian cities with significant lead emissions from mining and smelting. Previous screenings showed higher blood lead levels in Indigenous children. A study by Green et al. [64] tested thirty children aged 12–83 months from a Children and Family Centre over three half-days using capillary samples and the LeadCare II system. Among them, 40% had blood lead levels of ≥5 µg/dL, and 10% had levels of ≥10 µg/dL, with a maximum level of 17.3 µg/dL. Indigenous children had higher rates and mean levels than non-Indigenous children, a significant difference. Despite their small size, these results show that 40% of children have levels ≥5 µg/dL [64].

#### 2.3.7. Pb Pollution in Antarctica

McConnell et al. [65] analyze 16 ice cores, showing toxic lead pollution at the South Pole and across Antarctica by 1889, over 20 years before polar explorers. According to the paper, unlike the Arctic, where lead peaked in the 1970s, Antarctica had high lead levels from the early 1900s, similar to industrial peaks. Uniform timing and the Broken Hill lead signature across the continent suggest a single southern Australian source contributed to and still influences Antarctic lead pollution. About 660 tons of industrial lead have been deposited over 130 years from mid-latitude emissions, with regional and global circulation affecting aerosol levels. Lead pollution persists in Antarctica into the 21st century [65].

Ndungu et al. (2016) [66] report a decline in environmental Pb contamination after the near-global end of leaded gasoline in the 1970s. Snow and ice core data from Antarctica show that recent industrial Pb fluxes there have decreased. They present data on Pb levels and isotopic compositions in seawater and sediments from the Amundsen Sea continental shelf, including the Polynya. Their findings indicate that about 60–95% of Pb at sampling sites is natural, from weathering of Antarctic rocks. These natural Pb fluxes enter polynya waters via sediment resuspension or transport of sediment-laden glacial meltwaters [66].

Numerous sources of pollution described in this chapter lead to the manifestation of toxicity in living organisms. The toxicity of Pb is multifaceted and can affect all living organisms from bacteria to humans.

## 3. Pb Toxicity on Living Organisms

### 3.1. Pb Accumulation and Toxicity on Bacteria

**Figure 4:**
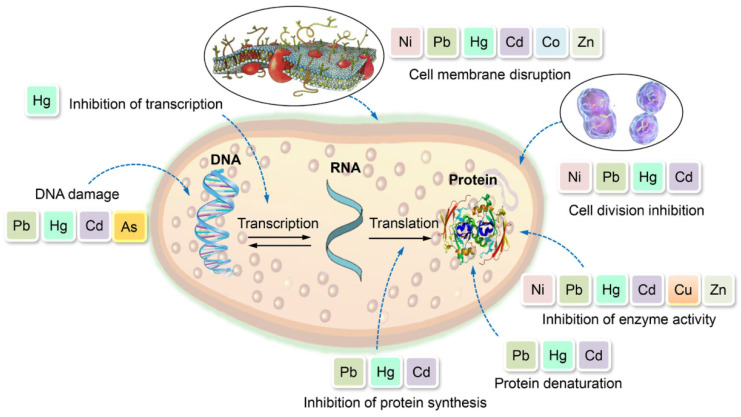
Toxicity of heavy metals on microorganisms [69].

Lead toxicity varies significantly among different strains, with some showing remarkable tolerance while others demonstrate high sensitivity. Gram-positive bacteria, such as *Bacillus* species, *Lactobacillus* strains (*L. acidophilus*, *L. rhamnosus,* and *L. plantarum*), and *Micrococcus* species, exhibit considerable lead resistance and removal capabilities through biosorption and bioaccumulation mechanisms [67]. Similarly, Gram-negative bacteria, including *Pseudomonas aeruginosa, Enterobacter cloacae,* and *Stenotrophomonas maltophilia*, demonstrate effective lead tolerance and detoxification abilities [67]. Examples of Pb-resistant bacteria isolated from contaminated sites include also *Acinetobacter junii Pb1*, *Bacillus subtilis X3*, *Delftia tsuruhatensis*, *Halomonas* sp., and *Pseudomonas aeruginosa N6P6* [68]. However, particular species, such as *Desulfovibrio desulfuricans* G20, show extreme sensitivity to lead, with complete growth inhibition at just 3 mg/L Pb(II) chloride. In contrast, *Thermus thermophilus* strain Samu-SA1 experiences reduced protein content and enzyme activity at much higher lead concentrations of 100–300 mg/L, or 100 times higher concentration [67] (Figure 4).

Bacterial adaptation mechanisms in Pb-contaminated environments involve biosorption, where Pb ions directly adsorb onto functional groups on bacterial cell surfaces. Among these functional groups are hydroxyl, amide, carboxyl, and phosphate groups, which are utilized as a detoxification strategy within the intracellular environment [70]. The efficiency of microbial-mediated immobilization depends on bacterial taxa and ambient environmental conditions, including pH, oxygen status, and metal concentrations [70]. Microorganisms employ key mechanisms, including immobilization of lead into insoluble precipitates through phosphate solubilization and microbially induced carbonate precipitation (MICP) [70]. Phosphate-solubilizing bacteria (PSB), widely distributed in soils, including the rhizosphere, can convert insoluble phosphorus compounds into reactive phosphate. The latter binds with Pb to form insoluble Pb-phosphate precipitates such as pyromorphite [Pb_5_(PO_4_)_3_X] [70]. Examples of PSB involved in Pb immobilization include *Bacillus subtilis*, *Pantoea* sp., *Enterobacter* sp., *Acinetobacter pittii*, *Serratia marcescens*, *Leclercia adecarboxylata*, and *Pseudomonas putida* [70]. MICP processes involve ureolytic organisms that produce carbonate (CO_3_^2−^) through urea hydrolysis, which then binds with Pb^2+^ to form insoluble lead carbonate (PbCO_3_) precipitates, including species such as *Sporosarcina* spp., *Viridibacillus arenosi*, *Enterobacter cloacae*, *Lysinibacillus sphaericus*, *Kocuria flava*, and *Staphylococcus epidermidis* [70]. Profound insights into the negative impact of Pb and other heavy metals on soil bacterial communities can be found in Satchanska’s monographs, which are based on her PhD thesis and subsequent research [3,18,22]. Unanimously, this research clearly demonstrates that some main subclasses of *Proteobacteria* have died out and have been replaced by other bacterial phyla.

### 3.2. Pb Accumulation and Toxicity on Fungi

Lead exposure induces significant morphological and physiological changes in fungal cells, including wrinkling, deformation, and the formation of crystalline precipitates such as lead hydroxyl apatite [71,72]. Lead toxic effects trigger oxidative stress responses and activate antioxidant defense systems, while simultaneously disrupting cellular membranes, DNA structure, and enzymatic functions [69]. Despite these adverse effects, metal-tolerant fungi maintain their bioremediation capacity through mechanisms including bioaccumulation, biosorption, and biotransformation, making them promising candidates for environmental cleanup applications [69,73].

The mechanisms of lead removal vary significantly with metal concentration and fungal species. At lower concentrations (25–50 mg/L), fungi primarily utilize intracellular accumulation, while at higher concentrations (75–300 mg/L), extracellular biosorption becomes dominant [74]. The biosorption process involves multiple functional groups, including O-H, C-H, N-H, C=O, and C-O groups, with peak biosorption capacity typically occurring on day 5 of incubation, reaching values up to 97.82 mg/g in *A. niger* [72]. *Sarocladium* strain Pb-9 showed maximum adsorption rates of 37.75% at 2000 mg/L concentration [71].

Multiple fungal species demonstrate remarkable tolerance to high lead concentrations, with *Aspergillus awamori* and *Aspergillus niger* strains showing outstanding tolerance at concentrations up to 10,000 mg/L [72]. *Verticillium insectorum* J3 survived and grew in medium containing 500 mg/L Pb(II), achieving approximately 90% removal rates at concentrations between 75 and 250 mg/L [74]. White rot fungi also demonstrate exceptional performance, with *Phanerochaete chrysosporium* removing over 90% of lead from 50 mg/L solutions and achieving up to 99% efficiency in industrial wastewater treatment [73].

### 3.3. Pb Accumulation and Toxicity on Edible Mushrooms

Wild edible mushrooms demonstrate significant capacity for lead bioaccumulation, with concentrations varying considerably among species and collection sites. Lead content in mushrooms typically ranges from 0.004 to 37.1 mg/kg dry weight, with *Umbilicaria esculenta* showing the highest accumulation potential at 12.3 ± 7.0 mg/kg d.w. [75]. Among commonly consumed species, *Suillus luteus* accumulated 2.21 ± 0.48 mg/kg d.w., while *Cantharellus cibarius* contained 0.424–1.01 mg/kg d.w. [76,77]. Transfer factors from soil to mushroom tissues range from 1.528% to 35.2%, with *Armillaria mellea* demonstrating particularly high bioaccumulation efficiency at 18.6% relative to underlying soils [78].

Most wild mushroom samples exceed the established regulatory limits for lead content, with mean concentrations surpassing the EU maximum level of 0.800 mg/kg dry weight (d.w.) for wild fungi [78]. However, when compared to the 3.0 mg/kg dry weight standard, naturally collected mushrooms from uncontaminated areas generally remain within acceptable limits [76]. The estimated daily intake of lead from mushroom consumption (2.51 × 10^−3^ to 3.12 × 10^−3^ mg/day) typically does not exceed provisional tolerable weekly intake levels, with health risk indices remaining below 1 for non-carcinogenic effects [75,79]. Despite relatively low daily intake levels, lead exposure from mushroom consumption presents concerning *carcinogenic* risks that exceed acceptable thresholds (10^−6^ to 10^−4^), with carcinogenic risk values ranging from 5.67 × 10^−4^ to 1.68 × 10^−3^ for adults [78].

### 3.4. Pb Accumulation and Toxicity on Plants

**Figure 5:**
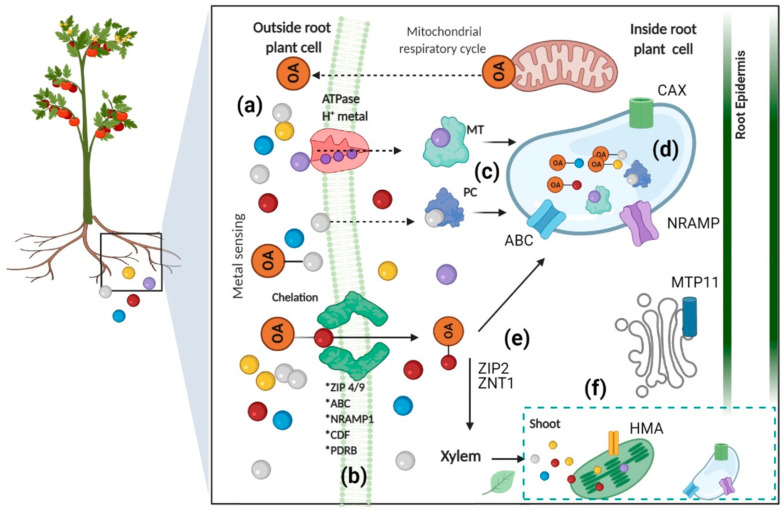
Visualization of lead uptake through the root system of plants, highlighting the roles of passive diffusion, facilitated transport, and active uptake. The figure shows how metals are absorbed and moved in plants at the physiological and molecular levels. Heavy metals like Pb, Cd, As (shown as colored circles) are taken up through root cells, where their presence or high concentration activate signaling pathways. These signals trigger plant defenses, including releasing mitochondrial-derived organic acids (OAs) that form complexes with metals outside roots (a), and transporting metals and metal–OA complexes into cells via transporters like ABC-type, ZIPs, cation diffusion facilitator (CDF), natural resistance associated macrophage protein 1 (NRAMP1), pleiotropic drug resistance (PDRB) and ATPase H+ metal (b), with asterisk (*) are noted the five different metal transporters. In the cytosol, metals can bind to chelators such as MTs and PCs (c), then be transported into vacuoles for storage or to other organelles like Golgi (d). Heavy metals may also move to the xylem via transporters like ZIP2 and ZNT1, then move to shoots (e). They can also be sequestered in vacuoles, Golgi (MTP11), or chloroplasts (HMA) through specific transporters (f). Organic acids are shown as orange circles [84].

Plants absorb Pb primarily through root systems via passive diffusion, facilitated transport, and active uptake mechanisms involving Zinc-Iron Permease (ZIP) and Natural Resistance-Associated Macrophage Protein (NRAMP) families (Figure 5) [80]. Soil physicochemical properties significantly influence uptake, with lower pH increasing Pb solubility and bioavailability [80,81]. The Casparian strip in root endodermis acts as a critical barrier, restricting Pb translocation from roots to shoots, resulting in more than 95% of absorbed Pb accumulating in root tissues [81]. This distribution pattern follows the sequence: roots > leaves > stem > inflorescence > seeds, with root vegetables typically showing the highest Pb concentrations [82]. Pb accumulation varies significantly among plant species, with hyperaccumulators like *Arabis paniculata*, *Isachne globosa*, and *Pogonatherum crinitum* capable of accumulating up to 1000 mg kg^−1^ Pb in tissue [83].

The World Health Organization has established a permissible limit of 2 mg/kg for Pb concentration in plants, while soil tolerance reaches 85 mg/kg [84,85]. However, Pb contamination varies significantly among agricultural systems, as demonstrated by lettuce grown in bioslurry-treated soils containing 16.7 mg/kg Pb, considerably exceeding the WHO/FAO permissible limit of 0.3 mg/kg for vegetables [86]. Fiber crops demonstrate variable accumulation patterns: Kenaf can accumulate up to 867.55 mg/kg Pb in shoots compared to 329.66 mg/kg in roots, while Hemp shows preferential root accumulation with concentrations reaching 38.2 mg/kg in roots versus 16.5 mg/kg in leaves [87].

Lead toxicity severely impairs plant growth and development, causing a 42% reduction in root growth and a 14–30% decrease in rice seed germination at 1 mM concentrations [82]. In maize cultivation, Pb exposure at 3000 mg/kg soil concentration caused a 20% reduction in plant height, a 15% decrease in fresh weight, and a 25% reduction in dry weight, alongside increased electrical conductivity (15.22%) and decreased leaf relative water content (62.32%) [88]. Lead exposure significantly affects stomatal structure and function, inducing stomatal closure in multiple species, including tobacco, white leadtree, black gram, and soybean, which contributes to reduced stomatal conductance and decreased transpiration rates [89]. Paradoxically, Pb can increase stomatal number and density in species such as sunflower and plantain, while causing ultrastructural changes in guard cells, including a decrease in guard cell diameter, and the accumulation of starch grains and plastid globules [89].

Pb exposure disrupts essential physiological processes, including photosynthesis, chlorophyll biosynthesis (particularly chlorophyll B), and ATP synthesis [80,82]. The metal interferes with nutrient uptake of essential cations (Mn^2+^, Zn^2+^, Fe^2+^, Ca^2+^, Mg^2+^) and induces oxidative stress through excessive production of reactive oxygen species (ROS), including H_2_O_2_, O_2_^2−^, and OH^−^ radicals [80,81]. Plants have evolved sophisticated defense mechanisms to counteract Pb toxicity, including activation of antioxidant defense systems comprising enzymatic antioxidants superoxide dismutase (SOD), catalase (CAT), peroxidase (POD), glutathione peroxidase (GPx), and non-enzymatic compounds (ascorbic acid, glutathione, carotenoids) [80,81]. Metal sequestration strategies involve the synthesis of phytochelatins and metallothioneins for Pb chelation, followed by vacuolar compartmentalization [90,91]. Cell wall binding through pectin-Pb complexes provides additional physical barriers, while specific genes, such as JcMT2a, JcPAL, and NtCBP4, have been identified as contributing to Pb tolerance in various species [90,91]. Advanced OMICS approaches have revealed complex molecular responses to Pb stress, including differential expression of 36 genes in maize roots and 181 proteins in *Rhoeo discolor* leaves exposed to Pb toxicity [92]. Epigenetic modifications play a crucial role in Pb tolerance, with DNA hypomethylation of transporter gene promoters (TaABCCs and TaHMA2) enhancing the metal sequestration capacity in wheat [93]. Genome-wide association studies have identified 4 quantitative trait loci (QTL) and 9 candidate genes related to Pb resistance in *Brassica napus*, while 21 QTLs were linked to Pb toxicity in rice grain [92].

### 3.5. Pb Accumulation and Toxicity on Animals

Pb bioaccumulation patterns vary significantly across animal species and tissues, with preferential accumulation in metabolically active organs. In invertebrates, lead accumulates in body tissues, nests (in social species), Malpighian tubules, and exoskeletons, where it may be temporarily stored as part of detoxification before molting [94]. Terrestrial invertebrates demonstrate heightened sensitivity to Pb exposure, experiencing detrimental effects at concentrations below current international permissible limits, including impaired fertility, developmental defects, and behavioral disturbances [94]. In marine fish, Pb preferentially binds to calcium-rich tissues such as bones and gills, while also accumulating in liver and kidney tissues through detoxification processes [95]. Unlike mercury, Pb generally does not biomagnify strongly through marine food webs and often exhibits trophic dilution, where concentrations decrease at higher trophic levels due to lower bioavailability and faster excretion rates [95]. Terrestrial food chains show distinct patterns of lead accumulation; for example, European raptors—especially obligate scavengers like Griffon Vultures—exhibit the highest mean Pb concentrations in liver and kidney tissues due to consumption of lead-contaminated prey [96]. Moreover, bone Pb concentrations exceeding 20 mg/kg dry weight are linked to clinical symptoms, with raptors and scavengers being particularly vulnerable to Pb-induced mortality, and hunting ammunition identified as a significant source of exposure [96,97].

The mechanism of lead toxicity in animals involves the production of free radicals that cause oxidative stress and damage to essential biological molecules, including enzymes, proteins, lipids, and DNA [98]. In cattle, chronic Pb exposure induces nephrotoxicity through increased lipid peroxidation and decreased antioxidant enzyme activity, with renal dysfunction occurring at concentrations as low as 10 g/dL [99]. Studies on wood mice from contaminated mining areas revealed that 93% of individuals at mine sites and 76% at private properties exceeded bone Pb thresholds (25 mg/kg dry weight) associated with kidney damage [100].

The ecological and public health implications of Pb contamination extend beyond individual animal toxicity to encompass population-level effects and concerns regarding food safety. Contaminated forage crops can lead to significant lead accumulation in grazing animals. For instance, cows grazing on polluted pastures showed mean Pb concentrations of 15 ± 2.6 µg/kg in milk, representing 75% of the maximum limit suggested by *Codex Alimentarius* [101]. Studies on farm ruminants showed that buffaloes consistently displayed the highest Pb levels in blood (up to 2.47 mg/L). At the same time, sheep exhibited the highest concentrations in wool hair samples (ranging from 1.05 to 2.35 mg/kg) [102]. In poultry, bioaccumulation primarily occurs in the kidneys, liver, reproductive organs, and lungs, with studies in various countries reporting varying tissue-specific concentrations [103]. The transfer of Pb through food chains poses significant human health risks, with studies in Middle Eastern countries frequently reporting Pb levels in red meat exceeding FAO and WHO permissible limits [104], emphasizing the critical need for comprehensive monitoring and mitigation strategies across agricultural and wildlife management systems.

Among all harmed organisms, the toxicity of Pb in humans is of greatest concern.

### 3.6. Pb Accumulation and Toxicity on Humans

Humans are primarily exposed to lead through the inhalation of polluted air and dust particles, ingestion of contaminated water or food, and, to a lesser extent, through dermal absorption [105]. Once absorbed, lead accumulates in the human body through bioaccumulation and biomagnification processes, with approximately 90% of absorbed lead stored in mineralized tissues, particularly bones and teeth, where it can persist for decades by displacing calcium in hydroxyapatite crystals [2,105]. The respiratory tract efficiently absorbs micron lead particles at approximately 69% efficiency, while gastrointestinal absorption occurs at 10–15% in adults and higher rates in children, primarily in the duodenum [2]. Children are particularly vulnerable due to higher absorption rates and greater susceptibility to lead’s toxic effects, as lead is not absorbed by their bones as efficiently as in adults, leaving more circulating in soft tissues [6].

**Figure 6:**
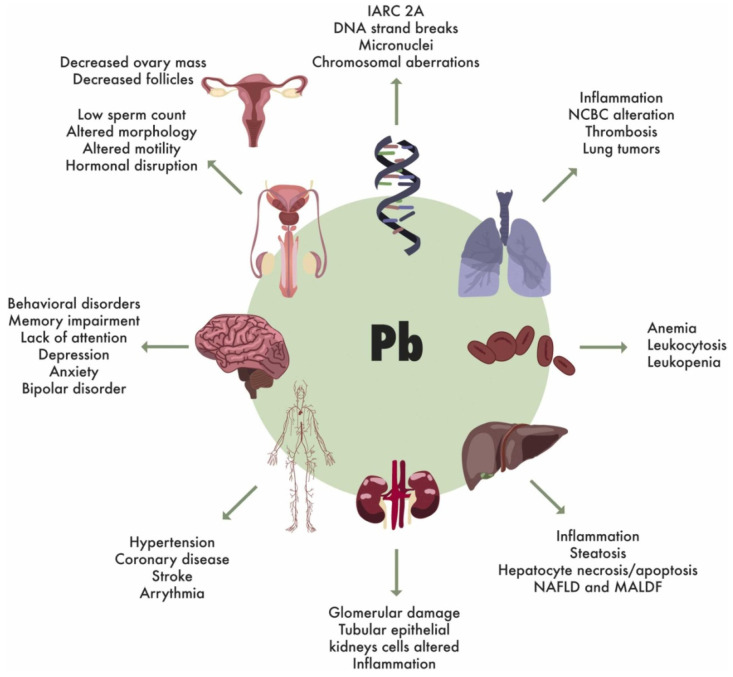
Lead (Pb) exposure induces toxic systemic effects [2].

Lead toxicity manifests systemically, affecting nearly every organ system, including the nervous system (causing neurotoxicity, cognitive impairment, and behavioral disorders), cardiovascular system (hypertension, increased risk of stroke, and coronary heart disease). Besides the above-mentioned afflictions, Pb also has adverse effects on the renal system (nephrotoxicity and chronic kidney disease), the reproductive system (reduced fertility, sperm abnormalities, and pregnancy complications), and the hematopoietic system (anemia and altered blood cell parameters) [2,6,106] (Figure 6). The World Health Organization has established that no level of lead exposure is considered safe for human health, with the international level of concern set at 10 μg/dL in blood. The Centers for Disease Control and Prevention in the USA have further restricted the permissible level of Pb in blood in children from 20 to 5 μg/dL. However, adverse effects can occur at even lower concentrations [106]. Lead’s persistence in biological systems, combined with its ability to cross critical physiological barriers, accumulate in various organs, and perturb protein function, makes it particularly hazardous to human health [107,108].

#### 3.6.1. Pb Distribution in the Human Body

**Figure 7:**
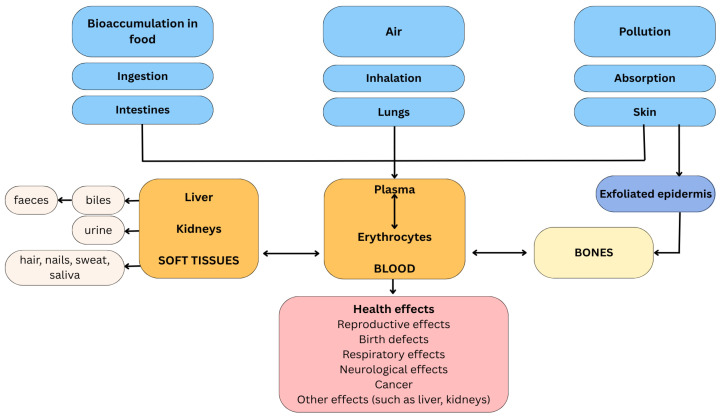
Pathways of lead accumulation and its effects on human health [7].

Understanding the distribution patterns of lead within the human body following different routes of exposure (Figure 7) is crucial for comprehensive risk assessment and the development of effective prevention strategies.

##### Inhalation

Lead exposure through inhalation represents a significant pathway for human contamination, particularly in occupational and urban environments. In these environments, anthropogenic sources, such as industrial processes—particularly those in sectors like welding and alloy smelting, as well as fossil fuel combustion and waste incineration—release these toxic elements into the atmosphere [109,110]. Once inhaled, lead is primarily absorbed from the respiratory system. However, it can also be absorbed from the digestive system when larger particles are swallowed, making inhalation a dual-route exposure mechanism [105,106]. In the blood, lead is primarily transported into erythrocytes by an anion exchanger. It interacts with intracellular proteins, such as aminolevulinic acid dehydratase (ALAD), which serves as a primary lead-binding site in red blood cells [105].

Lead concentrations in ambient air vary dramatically by location and source proximity, ranging from 0.05–0.1 µg/m^3^ in rural Mexican cities to 0.3–1.0 µg/m^3^ in major developing country cities [2]. Educational and office environments typically show low exposures of 0.02–0.274 µg/m^3^, while tobacco cafés demonstrate elevated levels of 0.7–0.9 µg/m^3^ [110]. Urban-industrial disparities are evident in Polish cities, where Pb particles smaller than 1 µm averaged 7.1 ± 0.46 µg/m^3^ in Warsaw, compared to 19 ± 1.4 µg/m^3^ in industrial Zabrze [109]. The highest concentrations occur near anthropogenic sources, with foundry areas exceeding 10 µg/m^3^ and Chinese lead-zinc smelters reporting annual averages of 8.05 μg/m^3^ Pb in air with winter peaks of 34.6 μg/m^3^ [2]. These concentrations are regulated by the WHO annual limits of only 0.5 µg/m^3^, EPA three-month limits of 0.15 µg/m^3^, and OSHA occupational limits of 50 µg/m^3^, with successful interventions demonstrated by Korea’s reduction from 0.34 μg/m^3^ in 1991 to <0.03 μg/m^3^ by 2018 following leaded gasoline prohibition [2].

##### Ingestion

Once ingested, Pb is absorbed through the gastrointestinal tract, with absorption rates varying significantly based on the chemical form and population demographics [111]. The gastrointestinal tract serves as the critical entry point for lead absorption from contaminated foods, where the metal undergoes oxidation and forms stable bonds to enzymes and protein molecules [111]. Following absorption, Pb is transported systemically via the bloodstream, primarily bound to erythrocytes, and subsequently distributes to various tissues and organs throughout the body [107]. Organic lead demonstrates substantially higher absorption rates, at approximately 90%, and is accumulated in bone tissue, where it replaces calcium ions, leading to reduced bone mineral density and prolonged retention. In comparison, inorganic lead absorption ranges from 40 to 50% in children, who exhibit markedly higher intestinal absorption capabilities compared to adults [111]. This enhanced absorption in pediatric populations is particularly concerning, given that children consume three times as much food relative to their body mass compared to adults [107,112].

International regulatory standards have established maximum permissible limits for lead in various food categories: 0.01 mg/kg in infant products and natural mineral waters, 0.02 mg/kg in milk and dairy products, and 0.1 mg/kg in fruits, vegetables, meat products, and wine [111]. However, monitoring studies have documented concerning exceedances of these limits, with apple samples from Ukraine and Kosovo, lettuce and red potatoes from Romania, milk and dairy products from Egypt, and various edible oils (olive, rapeseed, sesame, sunflower, corn, cottonseed, and soybean) from multiple countries all showing lead concentrations above regulatory thresholds (Table 4) [111].

Dietary fiber intake can modulate lead absorption by binding to Pb within the digestive tract, facilitating its excretion through feces and reducing systemic bioavailability [107]. Elimination of absorbed inorganic lead occurs primarily through renal excretion (approximately 75%) and fecal elimination (about 16%), though the metal’s slow excretion rate and prolonged retention time, especially in bone tissue, result in chronic bioaccumulation over extended periods [111].

##### Permeation

Dermal penetration of lead represents a significant yet under-studied exposure route that can contribute substantially to systemic lead burden [113]. While the outermost protective layer of the skin, the *Stratum corneum*, generally restricts large-scale penetration, traces of lead present in various sources can reach the circulatory system through skin appendages or via transcellular and intracellular pathways [114]. The vast majority of studies (92% of 24 study summaries) have reported detectable levels of dermal absorption of inorganic lead compounds, including both water-soluble and water-insoluble forms, with average calculated diffusion rates for animal and human skin data ranging from 10^−7^ to 10^−4^ mg cm^−2^ h^−1^ and permeability coefficient (Kp) values ranging from 10^−7^ to 10^−5^ cm h^−1^ [113]. These dermal exposures demonstrate significant potential impact, as they could potentially elevate blood lead levels by over 6 µg dL^−1^, representing more than 100% of the 5 µg dL^−1^ blood lead level associated with adverse health effects in adults [113]. Occupational exposure studies have documented measurable dermal lead concentrations, with the highest detected concentration of 6.91 ng/cm^2^ found in workers involved in stamping processes, corresponding to an Average Daily Dose of 301.59 ng/kg-day [115].

The bioaccumulation potential is particularly concerning given that some data suggest a “reservoir effect”, where lead may accumulate in skin layers and then act as a source for extended systemic exposure, highlighting the importance of comprehensive risk assessment that considers dermal penetration alongside traditional inhalation and ingestion pathways [113].

Among all heavy metals, Pb is a proven toxicant to nearly all human systems.

#### 3.6.2. Harmful Effects on Human Organs and Systems

**Figure 8:**
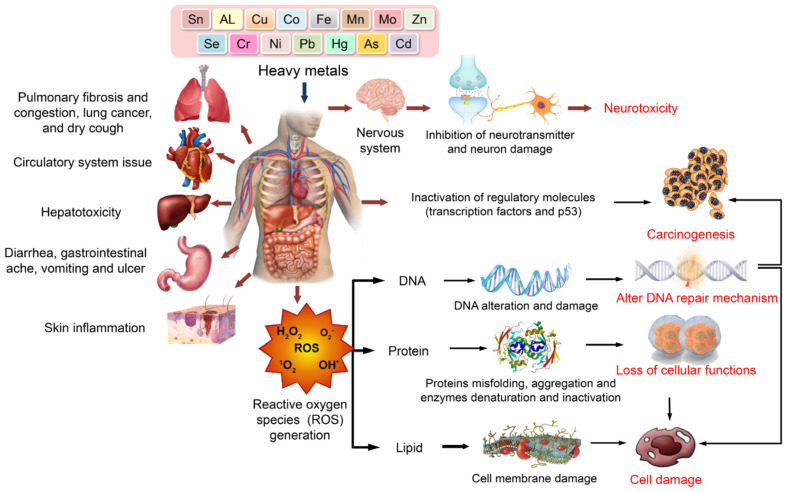
Oxidative stress and human organ toxicity following exposure to HMs [69].

Heavy metals exert toxic effects on various human organs and biological systems. Upon exposure, heavy metals can cause pulmonary, cardiovascular, hepatic, gastrointestinal, dermatological, and nervous system disorders. Central to their toxicity is the generation of ROS, which induces oxidative stress, leading to DNA damage, protein misfolding, lipid peroxidation, and membrane disruption. These molecular alterations contribute to neurotoxicity, carcinogenesis, impaired DNA repair, loss of cellular functions, and overall cell damage (Figure 8).

##### Effects on the Blood and Circulatory System

Chronic lead exposure is recognized as a significant risk factor for cardiovascular diseases (CVD), contributing substantially to global CVD deaths and disability-adjusted life-years (DALYs), with an estimated burden of 5.5 million deaths per year in 2019 [116]. Lead exposure affects the blood and circulatory system through multiple interconnected mechanisms, with approximately 99% of blood lead concentration bound to red blood cells [117]. The most extensively studied cardiovascular effect is elevated blood pressure, where a meta-analysis demonstrated that a 10 µg/g increase in bone lead was associated with a 0.26 mmHg increase in systolic blood pressure. In contrast, population studies have shown that declines in blood lead levels greater than 0.1 μg/dL result in marked reductions in systolic blood pressure [118,119]. Even low-level lead exposure around 2 μg/dL increases the risk of hypertension, peripheral arterial disease, and renal dysfunction, with estimated mortality relative risk from CVD being higher for subjects with blood lead levels above 5–9 μg/dL [117]. Participants experiencing the highest tertile of blood lead decline (>0.91 μg/dL) compared to the lowest tertile (<0.27 μg/dL) demonstrated a mean difference of −7.08 mm Hg in systolic blood pressure change [119]. Lead exposure results in increased blood pressure through oxidative stress, where chronic exposure intensifies the deactivation of nitric oxide by reactive oxygen species, leading to functional nitric oxide deficiency and compensatory upregulation of nitric oxide synthase [118].

Atherosclerosis serves as the major mediator of CVD from lead exposure, with studies showing that a 10 μg/L increase in blood lead is associated with a 7% increase in the risk of high coronary artery calcium score (≥100) in men, indicating significant coronary artery calcification [116]. Lead exposure contributes to ischemic heart disease, which accounts for the most significant proportion of lead-exposure-attributable CVD deaths and DALYs, and stroke, representing the second most significant cause of lead-related cardiovascular mortality [118]. The metal damages the blood vessel inner lining, promoting inflammation and plaque formation that narrows blood vessels, while also affecting cardiac structure through associations with decreased interventricular septum thickness [118,119].

At the cellular level, lead exposure interferes with cardiac conduction and contractility by reducing Na+/K+ ATPase pump activity. As a result, Pb affects myofibril phosphorylation and disrupts signaling pathways, while also promoting alterations in lipid metabolism, including increased triglycerides, lipid peroxidation, and decreased HDL cholesterol [117]. Lead exposure is also associated with anemia risk at high exposure levels, and the measurement accuracy of blood lead can be improved by correcting for hemoglobin or hematocrit levels, as lower erythrocyte volume fraction results in underestimated blood lead values [116].

##### Effects on the Reproductive System

Lead exposure during critical developmental periods, particularly puberty, causes lasting reproductive consequences in both males and females, including delayed sexual maturation, altered hormone profiles, and reduced reproductive capability [120]. Lead disrupts the hypothalamic-pituitary-gonadal axis in both males and females, interfering with essential hormone synthesis and release pathways that regulate reproductive function [121,122]. Lead exposure caused significant changes in serum sex hormone levels, with progesterone decreasing by 80.2% and testosterone by 49.9%, while estradiol increased by 69.8% in the highest dose group [120]. Furthermore, a meta-analysis found a statistically significant association between paternal lead exposure and congenital anomalies with a pooled odds ratio of 2.09 (95% confidence intervals: 2.09–3.35; *p* < 0.01) [122].

In males, lead exposure causes impaired spermatogenesis and disrupts the release of testosterone and luteinizing hormone, essential for sperm production and maturation [122]. Blood lead levels exceeding 40 µg/dL of Pb have been associated with significant impairments in male reproductive health, including reduced sperm count and concentration, decreased semen volume, diminished motility and vitality, and abnormal morphology. These highly negative consequences were demonstrated by both individual studies and a systematic review and meta-analysis [121,123]. Studies have shown a dose–response relationship between blood and seminal lead levels and worsening semen parameters, with even relatively low blood levels affecting male fertility [123].

In females, lead exposure adversely affects reproductive function by disrupting ovarian physiology, including impairments in follicle formation, oocyte maturation, ovulation, and *Corpus luteum* development. These disruptions contribute to reduced fertility, extended time to conception, and adverse pregnancy outcomes such as spontaneous abortion, stillbirths, and developmental abnormalities [121]. Additionally, epidemiological evidence links lead exposure to broader reproductive issues, including infertility, premature birth, and early menopause [120]. At the cellular level, lead induces oxidative stress in ovarian tissue by generating ROS, which damages DNA, lipids, and proteins, potentially causing genomic instability. This oxidative damage, combined with lead-induced inflammatory responses, may further contribute to pregnancy complications [121,122]. The molecular mechanisms underlying lead’s reproductive toxicity include the activation of endoplasmic reticulum stress-related signaling pathways, particularly the IRE1α-JNK signaling pathway, leading to increased follicular atresia and decreased ovarian reserves through apoptosis [121,122].

##### Effects on the Respiratory System

Adults are mainly exposed to lead-contaminated fine particulate matter and fumes through breathing, with respirable particles capable of deep lung penetration and deposition in the alveoli, readily passing the air–blood barrier for systemic distribution [124]. Prenatal and postnatal lead exposure has been associated with adverse respiratory health outcomes in children. It includes increased risk for atopic sensitization to common aeroallergens at 5 years of age and lung function deficits [125].

Several studies indicate that environmental lead pollution and lead exposure negatively impact the respiratory system in humans. In a study of 200 children aged 5–14 with bronchial asthma, higher blood lead levels were associated with increased asthma severity, as well as higher frequencies of eosinophilia and elevated IgE levels. Asthmatic children with elevated blood lead showed significantly higher rates of eosinophilia (66.7%) and increased total IgE (83.3%), along with more severe asthma, compared to children with normal blood lead levels. Another cross-sectional study, involving 1.788 children from the National Health and Nutrition Examination Survey (2005–2006), also found an association between blood lead levels and serum IgE levels, eosinophil counts, and asthma prevalence. Additionally, a higher frequency of asthma and respiratory symptoms was observed in industrial workers in the United Arab Emirates exposed to lead, compared to non-industrial workers, and a correlation between blood lead levels and asthma progression was reported among Caucasian Americans [126].

##### Effects on the Kidney System

Lead exposure poses significant nephrotoxic risks across multiple pathways, with the kidney serving as a primary target organ due to its role in lead excretion [127]. Chronic lead exposure results in accumulation in renal tissue, leading to continuous insults that manifest as proximal tubular dysfunction, chronic interstitial nephritis, and progressive chronic kidney disease (CKD), potentially culminating in end-stage renal failure [128]. Blood lead levels even below 5 µg/dL negatively affect kidney function, with the lowest level associated with longitudinal changes in estimated glomerular filtration rate (eGFR) documented at 2.2 µg/dL [129]. Elevated blood lead levels ≥1.5 µg/dL are significantly associated with increased CKD risk, maintaining significance after adjustment for confounders [130]. Meta-analysis reveals that chronic lead-exposed groups exhibit substantially higher blood lead levels averaging 25.6 µg/dL compared to unexposed populations, with elevated biomarkers including urinary N-acetyl-β-D-glucosaminidase (NAG), α_1_-microglobulin, β_2_-microglobulin, serum creatinine, and kidney injury molecule-1 (KIM-1) [128]. Pathological manifestations include glomerulosclerosis, tubular atrophy, interstitial fibrosis, and renal arteriosclerosis [127]. Lead exposure exhibits a dose–response relationship with kidney stone formation, where a 1-unit increase in urinary lead levels is associated with a 7% increased risk, and participants in the highest quartile have a 64% higher risk of kidney stones compared to those in the lowest quartile [127]. Among patients with advanced CKD (eGFR < 43 mL/min/1.73 m^2^), each 1 µg/g increase in tibial lead concentration correlates with 0.12 g/dL lower hemoglobin concentration and 23% higher odds of anemia, indicating lead’s role in CKD-related myelosuppression [131]. Systematically, lead enters proximal tubular cells through endocytosis, inhibits mitochondrial respiration, generates reactive oxidants, depletes intracellular glutathione, and triggers programmed cell death while binding to calcium-sensitive receptors and inducing hypercalciuria [129]. The nephrotoxic effects are mediated through oxidative stress, inflammation, activation of NF-κB, renin–angiotensin system activation, and macrophage recruitment, culminating in tubulointerstitial damage and progressive renal dysfunction [129,132].

##### Effects on the Bones

In bones, lead’s biological half-life lasts up to 30 years [133]. The metal exerts detrimental effects on bone health through multiple mechanisms, including its ability to replace calcium in hydroxyapatite crystals due to a higher affinity for bone sialoprotein than calcium [134], inhibition of vitamin D activation and dietary calcium absorption [135], and cytotoxic effects on osteoblasts, osteoclasts, and chondrocytes [133]. Studies demonstrate significant negative correlations between lead exposure and bone mineral density (BMD), with urinary lead levels showing correlation coefficients of −0.015 for total bone density and −0.019 for lumbar spine bone density in adults [134]. In children and adolescents, every 1 mg/dl increase in blood lead levels corresponds to BMD decreases of 0.011 g/cm^2^ at the total spine, 0.008 g/cm^2^ at the total femur, and 0.006 g/cm^2^ at the femur neck [135]. Lead exposure demonstrates site-specific effects, with lumbar spine BMD showing consistently negative associations while femoral BMD effects vary by study population [136]. Age and gender significantly influence lead’s impact on bone health, with stronger correlations observed in individuals ≥ 39 years and in women, particularly postmenopausal women, where lead may induce BMD decreases through estrogen level reduction [134]. Direct tissue analysis reveals significantly elevated lead levels in osteoporotic patients, with median bone tissue concentrations of 1.67 mg/kg in osteoporotic subjects compared to 0.57 mg/kg in healthy controls (*p* = 0.002), and corresponding negative correlations between bone lead levels and BMD of the total femur and femoral neck (rho = −0.33, *p* = 0.03) [133]. These findings demonstrate that lead exposure represents a significant risk factor for osteoporosis development, with effects detectable at low exposure levels, for children—even within current reference ranges [135].

##### Effects on the Liver and Intestines

The liver serves as a significant reservoir of lead among soft tissues, holding approximately 33% of the lead content, and chronic exposure results in statistically significant alterations in serum liver enzymes including increased levels of lactate dehydrogenase (LDH), aspartate aminotransferase (AST), anaplastic lymphoma kinase (ALK), alanine aminotransferase (ALT), and bilirubin, alongside decreased levels of total serum protein, albumin, and globulin [137]. Lead toxicity operates through multiple mechanisms, including oxidative stress generation, lipid peroxidation, and enhancement of inflammatory cytokines (IL-1β, IL-6, TNF-α), as well as interference with essential cellular processes. Even very low-dose exposure (median blood lead level of 0.9 μg/dL) can potentially cause organ injury [138,139]. Studies demonstrate that the proportion of advanced liver fibrosis significantly increases with blood lead level, with higher concentrations found in fibrotic groups (1.2 μg/dL vs. 0.9 μg/dL, *p* < 0.001), and blood lead levels remain an independent risk factor for advanced liver fibrosis even after controlling for confounding factors (OR = 1.249; 95% CI, 1.048–1.489; *p* = 0.013) [138]. The duration of lead exposure correlates significantly with the degree of liver fibrosis, with 26.7% of exposed individuals showing liver stiffness values above the significant fibrosis cut-off level [139]. Table 5 outlines selected hepatotoxic effects of lead exposure as observed in various in vivo studies utilizing animal models.

Regarding intestinal effects, lead causes gut dysbacteriosis by altering gut microbiota composition at the phylum, family, and genus levels, destroying gut physiological homeostasis through local oxidative stress and inflammation. Pb also increases gut permeability by reducing tight junction proteins expression, including peripheral membrane protein Zonula occludens-1 (ZO-1), Zonula occludens-2 (ZO-2), transmembrane proteins claudin-1 and occludin [141]. Lead exposure impairs intestinal absorption of essential nutrients, reducing glucose, glycine, lysine, and phenylalanine absorption, while disrupting the transport of divalent mineral elements like iron, calcium, and zinc by hijacking transporters such as Divalent Metal Transport 1 (DMT1) [141]. The gut-liver axis becomes compromised as dietary lead enters the portal vein through enterohepatic circulation, leading to disruption of hepatic lipid metabolism and dose-dependent increases in mRNA levels of genes related to fatty acid transport, β-oxidation, and triglyceride production [141].

##### Effects on the Central Nervous System

The neurotoxic mechanisms underlying lead’s deleterious effects involve complex neurochemical, molecular, and morphological changes that converge to influence neuronal activity and functional neural circuits [142]. Lead acts as a potent non-competitive antagonist of N-methyl-D-aspartate (NMDA) receptors; this inhibition leads to reduced brain-derived neurotrophic factor release, negatively impacting synaptogenesis and neuronal networks, ultimately resulting in impaired learning and memory abilities [143,144]. Lead’s ability to substitute for calcium ions facilitates its passage across the blood–brain barrier and disrupts calcium-dependent processes throughout the brain [145]. Additional mechanisms include oxidative stress generation through ROS production, mitochondrial dysfunction leading to decreased ATP levels, and interference with neurotransmitter systems. Particularly, it affects GABA and glutamate release, and disruption of myelin formation in the nervous cells’ axons [142]. Chronic exposure results in neuronal loss, reduced cortical thickness, deficient dendritic development, and decreased neurogenesis in the adult hippocampus [142]. Meta-analysis evidence indicates a significantly increased risk for malignant brain cancers associated with occupational lead exposure (pooled OR = 1.13, 95% CI: 1.04–1.24) [146].

Developmental neurotoxicity constitutes the most critical health effect, as prenatal and early postnatal exposure triggers irreversible alterations in CNS structure and function [142]. The developing brain demonstrates heightened vulnerability due to its rapid growth, immature blood–brain barrier, and high rates of cellular development [142]. Even low-level exposure below 10 μg/dL blood lead concentration produces significant cognitive impairments, with no evidence for a safe threshold identified [145]. Studies demonstrate that an increase in blood lead from <1 to 30 μg/dL associates with approximately 9 IQ point deficits, with the largest portion (~6 IQ points) occurring below 10 μg/dL [144]. An integrated analysis revealed a 6.9 IQ point decrement associated with increased concurrent blood lead levels from 2.4 to 30 μg/dL [145]. Lead exposure during elementary school age demonstrates maximal detrimental effects on IQ, resulting in deficits ranging from 5–7 points to up to 15 points in cognitive assessments [142].

##### Effects on the Immune System

The immune system demonstrates particular vulnerability to lead toxicity, with blood lead concentrations as low as 3.5 µg/dL causing significant immunological alterations in children [147]. Occupational exposure studies reveal dramatic effects, with shipyard workers showing median blood lead levels of 37.07 µg/dL—8.7-fold higher than controls at 4.3 µg/dL—and experiencing an 8.4% reduction in phagocytic activity, a 34% decrease in cytotoxic T cells (CD3+CD8+), and a 2.7-fold increase in regulatory T cells, alongside a 2-fold elevation in the IL-4/IFN-γ ratio indicating a shift toward humoral immunity [148]. At the cellular level, neutrophils accumulate the highest lead levels among immune cells and suffer vesicular damage through glutathione/multidrug resistance-associated protein (GSH/MRP) mediated sequestration, while CD4+ T cells, characterized as thiol-poor, experience severe ribosomal damage with reduced 18S rRNA levels [149]. Immunological perturbations involve oxidative stress mechanisms, inflammatory pathway activation including Mitogen-Activated Protein Kinase (MAPK), which is a type of serine/threonine-specific protein kinase involved in directing cellular responses, and NF-κB signaling (crucial intracellular pathway that regulates the expression of a wide range of genes), and result in immune dysregulation that increases susceptibility to infections, autoimmune diseases, and cancer [148,150].

Molecular mechanisms of Pb’s toxicity are still being studied as they are not yet completely unveiled.

## 4. Molecular Mechanisms of Pb Toxicity

### 4.1. Ion Mimicry and Cellular Disruption

Lead toxicity is driven by ion mimicry, wherein Pb^2+^ imitates essential divalent cations such as Ca^2+^, Mg^2+^, Zn^2+^, and Fe^2+^ due to similar biophysicochemical properties, allowing it to compete with or displace them at critical binding sites in proteins and enzymes, thereby disrupting their normal function [108,151]. Unlike these essential metals, however, Pb^2+^ cannot fulfill their physiological roles, making its substitution particularly harmful to mammalian cells. Pb^2+^ binds to functional sites normally occupied by calcium, magnesium, or zinc in key proteins such as calmodulin (calcium-binding protein), protein kinase C (play a crucial role in signal transduction pathways), troponin C (regulatory protein of striated muscle contraction,), and various synaptic proteins, with some Ca^2+^-binding proteins exhibiting even greater affinity for lead than for calcium [151]. In neurons, Pb^2+^ interferes with voltage-gated and receptor-operated calcium channels, impairing synaptic development and downstream signaling pathways, with potentially lasting neurotoxic effects [108].

Pb^2+^ competes with Zn^2+^ binding sites of several Zn^2+^ finger proteins (presence of zinc ions) and the NMDA receptor channel [151]. The metal also targets the displacement of Fe^2+^, particularly in the DMT1, which may facilitate Pb^2+^ transport and uptake. Pb^2+^ exposure increases Fe^2+^ content in the rat brain while decreasing ferroportin 1 (FP1), a Fe^2+^ efflux protein [151].

### 4.2. Mitochondrial Dysfunction and Energy Metabolism

Mitochondria represent primary targets of lead toxicity, with Pb^2+^ accumulating in the mitochondrial matrix and promoting imbalance in mitochondrial homeostasis [151]. Lead exposure remarkably reduces activities of mitochondrial respiratory chain enzyme complexes, especially complex II and complex III, impacting oxidative phosphorylation and decreasing ATP synthesis [152]. Studies have demonstrated that Pb exposure damages mitochondrial membrane potential, leading to decreased ATP production in rat proximal tubular cells, while chronic exposure causes mitochondrial edema in mice hepatocytes [153].

Lead disturbs intracellular calcium homeostasis through competition with Ca^2+^ for plasma membrane transport systems, including Ca^2+^ channels and pumps [152]. This leads to subcellular calcium redistribution, increasing Ca^2+^ levels in cytoplasm and mitochondria while decreasing endoplasmic reticulum (ER) Ca^2+^ [152]. Pb exposure increases expression of IP3R-1 and IP3R-2, enhancing ER Ca^2+^ release and subsequently increasing mitochondrial Ca^2+^ concentration [152].

**Figure 9:**
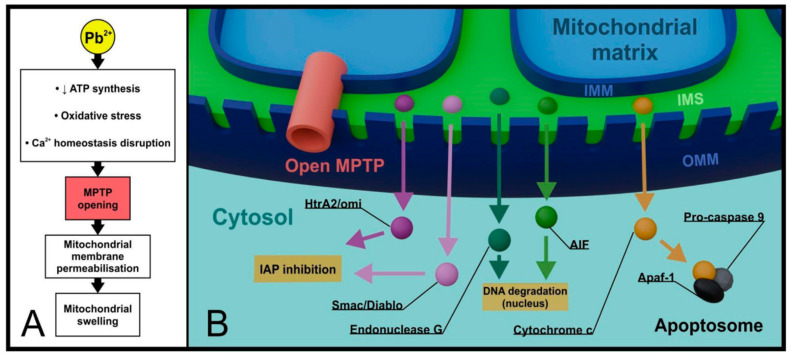
Lead and mitochondrial permeability transition pore involvement in late apoptosis and necrosis. Extensive metabolic changes in a cell exposed to lead result in decrease in ATP synthesis and in the opening of the mitochondrial permeability transition pores. The dramatically increased permeability of the outer mitochondrial membrane is accompanied by mitochondrial swelling (**A**) and a non-selective release of apoptosis-inducing factors from the intermembrane space into the cytoplasm (**B**) [154].

The pathological opening of the mitochondrial permeability transition pore (MPTP) represents a critical mechanism of lead-induced cellular damage (Figure 9) [152]. This abnormal MPTP opening results in a significant decrease in mitochondrial membrane potential, ultimately leading to cell death through apoptosis [152]. Lead exposure alters the expression of MPTP components, including the voltage-dependent anion channel (VDAC), adenine nucleoside translocator protein (ANT), and cyclophilin-D (Cyp-D), while significantly increasing the expression of the pro-apoptotic protein Bax and decreasing the expression of the anti-apoptotic proteins Bcl-2 and Bcl-xL [152].

### 4.3. Oxidative Stress and Antioxidant Depletion

Lead exposure generates ROS and reactive nitrogen species (RNS) either directly or indirectly within mitochondria [152]. The brain demonstrates particular susceptibility due to high oxygen consumption, low antioxidant defenses, abundance of polyunsaturated fatty acids, and presence of redox-active metals like Fe^2+^ [151].

Lead significantly inhibits the mitochondrial antioxidant system, including activities of GPx, SOD, CAT, and glutathione (GSH) [152]. Pb demonstrates high affinity for reactive -SH groups of GSH, effectively decreasing GSH levels [106]. The metal inhibits antioxidant enzymes by competing for essential metal cofactors like Zn^2+^ and Cu^2+^ [151].

Lead contributes to free radical generation through pro-oxidative effects of δ-aminolaevulinic acid (δ-ALA), which accumulates due to Pb^2+^-induced inhibition of the enzyme ALA dehydratase (an enzyme known also as porphobilinogen synthase that plays a crucial role in the biosynthesis of heme, chlorophyll, and vitamin B_12_) [151]. This leads to increased levels of oxidative stress markers, including malondialdehyde (MDA) and H_2_O_2_ concentrations [106]. Lipid peroxidation and protein carbonylation serve as significant indicators of oxidative stress under Pb exposure [152].

### 4.4. Neuroinflammation and Immune Response

Lead exposure stimulates neuroinflammation through activation of microglia and astroglia with subsequent release of inflammatory mediators [151]. Pb-induced mitochondrial damage promotes inflammation, potentially via TLR4-stimulated signaling cascades [152]. In general, TLRs are an important family of pattern recognition receptors (PRRs) that recognize exogenous and endogenous pathogens, heat shock proteins, and different components of the extracellular matrix. MPTP opening releases Mitochondrial Damage-Associated Molecular Patterns (MTDs), including the circular mtDNA, ATP, cardiolipin, and formyl-peptides, which act as DAMPs and activate pattern recognition receptors like TLR4 and TLR9 [152]. Cardiolipin, 1,3-bis(sn-3′-phosphatidyl)-sn-glycerol, is an important component of the inner mitochondrial membrane responsible for cytochrome-C-Oxidase quaternary structure, while formyl-peptides, also located in mitochondria, play a role in the initiation of inflammation by activating the formyl-peptide receptor (FPR). Astroglial and microglial activation also associates with elevated levels of NADPH oxidase (NOx), the main non-mitochondrial ROS source activated by Pb^2+^ [151]. Lead exposure triggers assembly and activation of NLRP3 inflammasomes (multiprotein complexes that play a pivotal role in regulating the innate immune system and inflammatory signaling), leading to pyroptosis through pro-caspase-1 cleavage and maturation of IL-1β and IL-18 [152]. Pyroptosis is a highly inflammatory-driven, caspase-1-dependent programmed lytic cell death that is often caused by the invasion of an intracellular pathogen.

The TLR4-MyD88-NF-κB axis represents a significant pathway in Pb-induced inflammation [152]. Lead increases expression of TLR4 and MyD88 (Myeloid differentiation 88 protein, with an important role in the immune system), activating downstream molecules including IRAK-4, IRAK-1 (IRAK–interleukin-1 receptor-associated kinase), involved in innate immune responses, and TRAF6, ultimately leading to NF-κB (a key modulator of innate and adaptive immune responses) activation [152]. Activated NF-κB and p38 MAPK (Mitogen-Activated Protein Kinase) pathways translocate to the nucleus, upregulating the expression of cytokines, including TNF-α, IL-1, IL-6, IL-8, IL-10, and IL-18, and provoking chronic inflammation [106,152].

### 4.5. DNA Damage and Genotoxicity

Lead induces genotoxic effects primarily through oxidative stress and inhibition of DNA repair mechanisms [155]. The accumulation of ROS impairs energy metabolism and causes various DNA alterations, including fragmentation, rearrangements, deletions, and point mutations [108]. Additionally, lead can directly interact with nuclear proteins and DNA, leading to site-specific damage and further ROS production [108]. One contributing factor is the accumulation of δ-ALA, which promotes ROS generation and oxidative DNA damage, such as the formation of 8-hydroxy-2′-deoxyguanosine (8-OHdG)—a widely recognized biomarker of oxidative stress that shows a positive correlation with lead exposure [155].

Lead interferes with DNA processing and repair enzymes by substituting essential metals like calcium and zinc [155]. Studies demonstrate altered expression of DNA repair genes due to Pb toxicity, with significant decreases in expression of genes involved in base excision repair (BER), nucleotide excision repair (NER), and double-strand break repair [155]. Specific genes including OGG1 (encodes the enzyme 8-oxoguanine DNA glycosylase responsible for the excision of 8-oxoguanine, a mutagenic base byproduct which occurs as a result of exposure to reactive oxygen), APE1(encodes multifunctional protein involved in DNA repair), and XRCC1 (BER pathway), XPD (xeroderma pigmentosum group D (XPD) protein with a function in nucleotide excision repair of DNA damage caused by UV radiation) and POLD1 (NER pathway), and BRCA1 (homologous recombination) show down-regulation following Pb exposure [155].

### 4.6. Epigenetic Modifications

Lead acts as an epigenetic toxicant by altering DNA methylation, histone modifications, and the expression of non-coding RNAs [156]. DNA methylation dysregulation involves changes in 5-methylcytosine (5 mC) levels and abnormal methylation of genes involved in neurodevelopment, cognitive function, and cell cycle regulation. Lead exposure alters the expression levels of DNA methyltransferases (DNMTs), including Dnmt1, Dnmt3a, and Dnmt3b, as well as demethylases and methyl-binding proteins such as MeCP2 [156].

Histone modifications include methylation and acetylation of N-terminal tails of histones H3 and H4 [156]. Lead exposure causes significant differences in levels of post-translational histone modifications, specifically H3K9/14ac and H3K9me3 (acetylation of specific lysine residues), with decreased H3K27me3 levels observed in the hippocampus of lead-exposed rodents. Lead treatment results in HDAC2 (Histone Deacetylase 2) upregulation and Ac-H3K9 (modification of Histone H3, indicating the acetylation at the 9th lysine) downregulation, with HDAC2 considered essential for regulating lead-induced neurotoxicity [156].

MicroRNA alterations include upregulation of miR-520c-3p, miR-211, miR-148a, and miR-572 in occupationally exposed Chinese workers [157]. MiR-520c-3p, miR-211, miR-148a, and miR-572 are closely related to cancer. MiR-520c-3p, a novel suppressor of lung adenocarcinoma, was recently found to be upregulated in various tumors, including gliomas. MiR-148a acts as a tumor suppressor and shows great potential in renal cancer therapy, and miR-572 (both squamous cell carcinomas of the head and neck and ovarian cancer). Studies show significant upregulation of miR-155 and miR-221, with functional analysis predicting targets involved in cellular pathways including cell differentiation, development, and apoptosis [157].

### 4.7. Autophagy and Cell Death Pathways

Lead triggers autophagy, particularly mitophagy (selective degradation of damaged mitochondria), via mitochondrial pathways [152]. Pb affects mitochondrial dynamics by altering expression of proteins including MFF (Mitochondrial fission factor controlling the division of mitochondria), Drp1 (dynamin-related protein 1, tightly regulated to clear the damaged mitochondria), MFN1 (mitofusin, functions as a tumor suppressor gene, activating the fusion process to depress the metastasis and invasion in vitro and in vivo), MFN2, and OPA1 (crucial regulators of the mitochondrial dynamics), increasing fission factors while decreasing fusion factors [152]. Lead exposure accelerates mitophagy via the PINK1/Parkin pathway (an autophagy machinery that cleans defective mitochondria), causing mitochondrial depolarization and accumulation of uncleaved PINK1 (a kinase that functions as a mitochondrial damage sensor) on the outer mitochondrial membrane [152]. Lead exposure triggers non-apoptotic cell death pathways, including necroptosis—via upregulation of RIPK3 (lipid metabolism regulator contributing to inflammation and carcinogenesis in non-alcoholic fatty liver disease) and MLKL (Mixed lineage kinase domain-like pseudokinase, an executioner of necroptosis) in olfactory cells. In addition, lead induces ferroptosis by increasing free iron levels, downregulating GPX4 (Glutathione peroxidase, a selenonzyme that plays a critical role in maintaining oxidative homeostasis)*,* and altering SLC7A11 (L-cystine/L-glutamate transporter across the cell membrane) expression in the most commonly used cell line in neuroscience research, especially for Parkinson’s, cell line–PC12 [153].

Organisms affected by Pb-rich environments adapt through various defense and resistance mechanisms.

## 5. Pb Resistance Mechanisms: Summary

**Figure 10:**
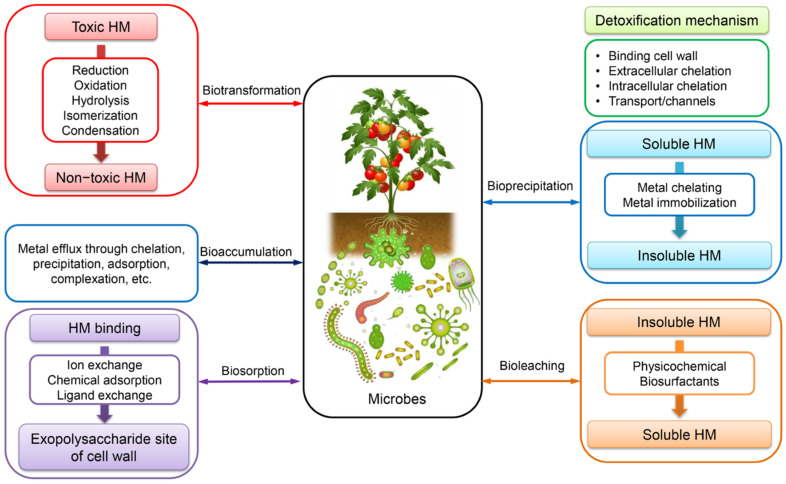
Microbial remediation mechanisms against heavy metals, including Pb [69].

Lead is lethal to bacterial cells even at small concentrations [67]. At high concentrations, Pb inhibits cell division, disrupts cellular respiration, and alters the morphology and membrane integrity of bacterial cells [158]. In response to this toxicity, organisms have developed sophisticated defense mechanisms (Figure 10) mediated by heavy metal resistance genes (HMRGs) [68]. Resistance is typically determined by measuring the minimum inhibitory concentration (MIC), with strains showing high Pb resistance often having an MIC greater than 1 mM [70].

### 5.1. Efflux and Active Transport Systems

Bacteria have evolved sophisticated resistance mechanisms to counteract lead toxicity, employing multiple strategies for survival in contaminated environments. Efflux systems play a central role in microbial defense against lead toxicity in Pb/Zn tailings, with membrane-bound enzymes called P-type ATPases such as ZntA actively exporting Pb^2+^ ions from the cytoplasm to the external environment through ATP hydrolysis, thereby maintaining cellular homeostasis under metal stress [158,159] PIB-type ATPases mediate the efflux of lead, as observed in *Pseudomonas* species, which actively extrude metal ions to withstand heavy metal stress [67]. The *zntA* gene, encoding a P-type ATPase, is particularly significant and highly expressed in both rhizosphere and non-rhizosphere soils of milltailings regions [159]. Under high lead exposure, the *pbrTRABCD* gene cluster becomes specifically activated, with elevated expression of genes such as *pbrB* (encodes fusion protein PbrBC), *pbrT* (encodes Pb-uptake protein), and *pbrD* (encodes a Pb-binding protein, involved in sequestration and detoxification within the cell), which encode proteins involved in lead transport and sequestration. In parallel, other efflux systems—particularly RND family transporters and ABC-type pumps—utilize ATP hydrolysis or proton gradients to extrude toxic Pb^2+^ ions, further contributing to microbial resilience in contaminated environments [68,160].

Plants utilize active efflux mechanisms through ATP-dependent transporters at the root level to limit heavy metal uptake, creating competition with other nutrients for absorption sites (Figure 10) [87]. Once heavy metals enter the plant cytosol, they can be transported out of cells or restricted to vacuoles through sequestration involving transporter families like ABC (transport of sugars, amino acids, and metals), CDF (transport of divalent cadmium, zinc, and cobalt), HMA (heavy-metal-associated domain), and NRAMP (Natural Resistance-associated Macrophage Protein), transport of divalent iron and manganese) [90].

### 5.2. Metal Chelation and Sequestration

**Figure 11:**
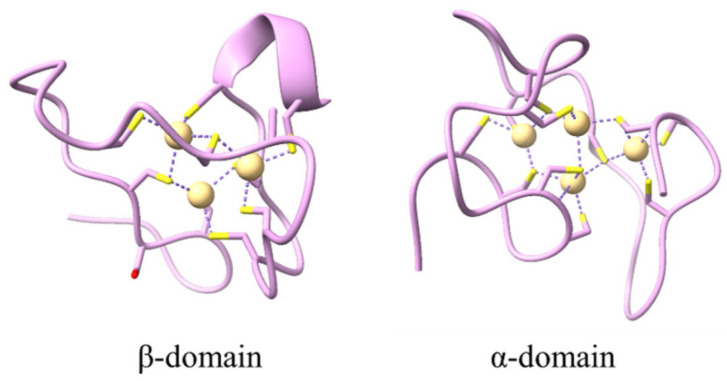
The NMR structure of the α-domain and β-domain of rat MT-2. The α-domain (purple) has four (yellow) metal ion binding sites, and the β-domain (purple) has three (yellow) metal ion binding sites [161].

Metal sequestration mechanisms play crucial roles in lead resistance through intracellular binding and precipitation strategies. Metallothioneins are cysteine-rich proteins that sequester heavy metals through thiol groups, forming non-toxic complexes in bacterial, yeast, plant, and animal cells, including human cells (Figure 11). Cysteine-rich metallothioneins effectively trap toxic heavy metals, including Pb, allowing bacteria to tolerate high concentrations by forming non-toxic complexes within the cytoplasm [68]. Glutathione acts as an alternative chelator, scavenging and detoxifying lead through its thiol group, while metallochaperones assist in transporting metal ions within cells to minimize toxic impact [68]. Some bacterial species, including *Bacillus*, *Staphylococcus aureus*, *Shewanella*, *Providencia*, and *Vibrio harveyi*, employ precipitation mechanisms by sequestering lead as phosphate salts, forming intracellular precipitates that reduce bioavailability [68,160]. For example, the genetically engineered *Pseudomonas putida* strain 15420352 overexpressing metallothioneins has shown enhanced capability to capture and immobilize Pb and Hg in wastewater, proving effective in pilot-scale bioreactors for treating industrial wastewater from mining and battery production [158]. Under lead stress, microorganisms adjust the expression of metallothionein genes by utilizing metal-sensing proteins, metal-responsive transcription factors, metal-binding proteins, and phosphorylation mechanisms to cope with fluctuating metal ion concentrations [159].

Siderophores, produced by bacteria such as *Alcaligenes eutrophus* and *Pseudomonas aeruginosa*, can reduce Pb toxicity. *P. aeruginosa* produces specifically pyoverdine and pyochelin, which can block Pb absorption [158]. Enhanced siderophore production serves as a lead-resistant mechanism. It is typically produced to promote plant growth in iron-deficient environments while influencing heavy metal bioavailability [67].

Plants employ phytochelatins (PCs) as specialized metal-binding peptides under heavy metal stress. PCs are high-affinity ligands that bind to metal cations, including Pb^2+^, to immobilize them, preventing interference with essential biochemical pathways and cell signaling [90]. Plants also synthesize organic acids to bind free metal ions, thereby minimizing toxic effects [90].

### 5.3. Biosorption and Surface Binding

In bacteria, biosorption involves the binding of metal ions to bacterial cell walls and their entrapment in extracellular capsules or exopolysaccharides (EPS), with functional groups such as hydroxyl, amino, amide, and phosphate playing key roles in the adsorption of heavy metal ions [67,159]. *Bacillus megaterium* plays a crucial role in detoxifying Cd and Pb through the production of EPS [38]. Biofilms, composed of EPS, create protective niches for bacteria and bind heavy metals, thereby lowering their toxicity and bioavailability [158]. The binding of Pb^2+^ has been noted at the interface of *Burkholderia cepacia* biofilms and hematite (Fe^3+^ oxide) [68].

In fungi, cell surface binding and extracellular biosorption serve as the primary mechanisms for removing Pb(II) at higher concentrations, with extracellular polymeric substances or metabolic products coating the cell surface and facilitating lead removal through mechanisms such as surface binding, ion exchange, or precipitation [74]. Fourier transform infrared spectroscopy analysis identified various functional groups involved in biosorption, including O-H and N-H bonds from proteins, C-H stretching vibrations from hydrophilic lipid molecules, and protein components like Amide I (C=O stretching) and Amide II (NH_2_ deformation) peaks [71].

Plant cell walls act as physical barriers where bivalent metal cations such as Pb^2+^ bind to carboxyl groups in pectin, restricting heavy metals to cell walls and preventing cellular entry [90]. Lead accumulates in the cell walls of epidermal cells in certain plants [90].

The sorption capacity of specific microorganisms is presented in Table 6.

### 5.4. Bioaccumulation and Intracellular Sequestration

Bioaccumulation refers to the accumulation of metal ions within cells in less toxic forms or as intracellular precipitation, often being dependent on metabolic activity [67]. Intracellular sequestration involves complexation and storage of metal ions within the bacterial cytoplasm [158].

In fungi, intracellular accumulation plays a vital role in Pb(II) removal, particularly at lower concentrations (25–50 mg/L^−1^). *Verticillium insectorum* J3 can take up and accumulate Pb(II) ions inside fungal cells [74]. Scanning Electron Microscope Observation of *Sarocladium* Pb-9 indicated that the Pb element was contained within the mycelium after treatment with Pb(NO_3_)_2_ [71].

### 5.5. Precipitation and Biotransformation

Lead precipitation represents an effective detoxification strategy across multiple organism types. Sulfate-reducing bacteria can precipitate Pb, and precipitation can be catalyzed by enzymes such as phosphatase enzyme (PbrB) [67]. The mechanism of MICP precipitates Pb in its carbonate form, serving as a resistance mechanism by sequestering Pb^2+^ and other toxic metals, thereby protecting microbial cells [70].

Fungi can facilitate heavy metal detoxification through biotransformation and precipitation of Pb^2+^ as insoluble compounds, including lead hydroxyl apatite, often aided by pH increase and metabolic activity [71,74].

### 5.6. Morphological Adaptations

Morphological alterations play a critical role in microbial resistance to heavy metal stress. Under lead (Pb) exposure, bacteria often exhibit changes in cell shape, size, and membrane integrity as part of their adaptive response, demonstrating specific evolutionary adaptations and regulatory responses to metal stress [158]. *Pseudomonas* species, in particular, demonstrate advanced survival strategies, including morphological differentiation, biosurfactant production, and the expression of stress-responsive proteins [159]. *Proteobacteria* are noted to carry most heavy metal resistance genes, with metal-resistant taxa including *Afipia, Bradyrhizobium*, *Sphingomonas*, and *Miltoncostaea* showing particular adaptations to lead-rich environments [158]. *Pseudomonas* S8A employs comprehensive strategies, including morphological differentiation, surfactant production, and specific protein expression for lead resistance [158].

Under lead stress, fungi exhibit morphological adaptations, including distorted, aggregated hyphae, hollow pellets with entangled structures, and deformed, wrinkled spores with surface deposits, which enhance metal tolerance and extracellular adsorption capacity [71,74].

### 5.7. Genetic Regulation and Molecular Mechanisms

Bacterial tolerance involves genetic mutations targeting genes responsible for regulating metal ion absorption, efflux, and storage. Horizontal Gene Transfer (HGT) significantly contributes to metal tolerance by facilitating the rapid spread of resistance genes across bacterial species through mobile genetic elements like plasmids and transposons [158].

Heavy metals can directly trigger the transcription of resistance genes, thereby initiating detoxification and tolerance responses [159]. Inorganic ion transport and metabolism play a crucial role in maintaining bacterial homeostasis and mediating heavy metal transport by regulating intracellular ion levels and metal metabolism [159].

## 6. Conclusions

Lead contamination represents one of the most pervasive and persistent environmental health challenges, affecting virtually every biological system through complex molecular mechanisms involving ion mimicry, mitochondrial dysfunction, oxidative stress, and DNA damage. With an estimated 900,000 deaths annually and no safe exposure threshold, lead poses irreversible neurodevelopmental risks in children and cardiovascular consequences in adults, persisting in bone tissue for decades after exposure. While organisms have evolved resistance mechanisms, including efflux systems, metal chelation, and biosorption, and promising remediation technologies such as nanotechnology applications, microbial bioremediation, and phytoremediation continue to advance, prevention through source control remains the most effective approach. The ultimate goal must be the elimination of lead exposure to the greatest extent possible, requiring sustained research, technological innovation, policy development, and international cooperation to address this preventable global health crisis and protect current and future generations from one of humanity’s oldest and most persistent environmental toxins.

## Figures and Tables

**Table 1:** Physical and chemical properties of Pb [1].

Property	Value
Atomic number	82
Atomic weight	207.2 u
Atomic radius	180 pm (Empirical)
Electronic configuration	[Xe]6s^2^4f^14^5d^10^6p^2^
Melting point	327.46 °C
Boiling point	1749 °C
Density at 20 °C	11.342 g/cm^3^
Reduction potential Pb^2+^ + 2e^−^ → Pb(s)	−0.126 V
Heat of fusion	4.77 kJ/mol
Heat of vaporization	179.5 kJ/mol
Electronegativity (Pauling scale)	2.33
First ionization energy	7.417 eV
Second ionization energy	15.03 eV
Isotopes	^204^Pb, ^206^Pb, ^207^Pb, ^208^Pb

**Table 2:** Lead Concentration (mg/kg) in Soil, Dust, and Marine Samples from Selected Global Regions. Table is optimized after [9,32,33].

Sample	Lead Concentration Range
Urban soil, Klang district, Selangor	52.7
Soil from the mango plantation area, Perlis	0.4
Surface soils from iron ore mining sites, Kuala Lipis, Pahang	63.5–72.5
Grassland, arable land, forest, wasteland, Malopolska, Poland	3–586
Average concentration of heavy metals in the Earth’s crust	12.5
Dust fall particles, Zarand, Iran	1.01
Road dust, Delhi city, India	128.7
Red Sea (North), Gulf of Aqaba	96.67
Red Sea (North), Hurghada City	53

**Table 3:** Maximal permissible limits of Pb. These levels are often based on the route of exposure and can vary significantly by country and land use.

Metric	Pb	Ref.
Food	0.01–3 mg/kg	[41]
Drinking water	5 µg/L	[42]
Soil	50–300 mg/kg	[43]
Air	0.5 µg/m^3^	[44]

**Table 4:** Measured lead levels in selected products. The table is optimized after [111].

	Food Type	Country	Concentration (μg/kg or μg/L)	Year
F R U I T S	Grape white varieties	Croatia	0.001–0.021	2008
	Grape red varieties	Croatia	0.002–0.039	2008
	Banana	Bangladesh	3	2016
	Mango	Bangladesh	642	2016
	Apples	Kosovo	1490–2170	2019
	Apples	Ukraine	1347–3886	2021
V E G E T A B L E S	Lettuce	Romania	820–2220	2021
	Tomato	Romania	0.7–0.8	2023
	Potato	China	67	2009
	Potato	Bangladesh	7	2016
	White potato	Romania	300–400	2021
	Red potato	Romania	370–1030	2021
	Onion	Romania	160–180	2021
	Carrot	Romania	540–940	2021
	Beans	Romania	80–520	2021
	Soybean	Monte Carlo	33–70	2022
	Grain, maize	China	20–13	2015
M E A T S	Pork meat products	Italy	220–380	2020
	Pork	Italy	0.024	2020
	Bacon	Romania	580	2014
	Ham	Romania	650	2014
	Salami	Romania	210	2014
	Sausages	Romania	820	2014
	Red meat	Asia	605–1435	2023
	Red meat	Africa	840–1094	2023
	Beef	Italy	0.019	2020
	Mutton meat	China (Beijing)	128	2019
D A I R Y	Milk	Monte Carlo	550	2023
	Milk	Turkey	0.85	2023
	Milk	Tanzania	263	2023
	Raw cow milk	Turkey	16.7	2012
	Raw cow milk	Egypt	101.6	2023
	Sheep and goat milk	Italy	0.002	2020
	Milk and dairy products	Egypt	0.044–0.751	2014
	Full-fat UHT milk	Cyprus	2.66	2021
	Full-fat yogurt	Cyprus	3	2021
	Halloumi cheese	Cyprus	35.3	2021
O I L S	Corn oil	Iran	99	2020
	Olive oil	London	143	2022
	Olive oil	Pakistan	4.285	2022
	Rapeseed oil	China	1.960	2016
	Rapeseed oil	Poland	56	2017
	Coconut oil	London	158	2022
	Sesame oil	Pakistan	4.005	2022
	Sesame oil	Korea	36	2019
	Sunflower oil	London	274	2022
	Sunflower oil	Iran	99	2020
	Flaxseed oil	Korea	25.7	2019
D R I N K S	Beer	Ethiopia	6	2022
	Beer	Brazil	13–33	2005
	Muscat Ottonel	Romania	2.5–632	2017
	low-alcoholic Muscat Ottonel	Romania	67–575	2017
	White wine	Croatia	30	2008

**Table 5:** Effect of lead on the liver. The table is optimized after [140].

Effect	Concentration	Exposure Time	Biological Models	Mode of Action	Outcome of Treatment
Oxidative stress	Lead acetate (Pb 0.2%)	5 weeks	Rat	Upregulating the transcription process of the cyclooxygenase-2 gene	oxidative stress, lipid peroxidation
Ultrastructural changes	0.13% lead acetate	4–8 weeks	Adult albino rats	Megalocytosis complex III of the respiratory chain affected	nuclear pyknosis, juxtanuclear inclusion bodies
Cholesterol functions of the liver	Lead acetate (500 mg Pb/L)	10–11 weeks	Male Wistar rats	Inhibition of the activity of HMGR and decrease in the expression of cholesterol 7 alpha-hydroxylase (CYP7A1) genes	reduction in metabolism of cholesterol, an increase in plasma cholesterol levels
Metabolic functions	Lead acetate or lead nitrate (20 mg/kg)	4 weeks	Swiss albino male mice	Reduced enzymatic activity of glucose-6-phosphatase (G6PASE)	pyruvic acid content was increased, disruption in glycogen-related mechanisms
Hepatic hyperplasia	Lead acetate trihydrate	4–52 weeks	Wistar Albino Rats	Increase in the activity of DNA polymerase-β, Protein kinase C alpha (PKC-α) overexpression, suppression of the mRNA of the CYP1A2 gene, increased production of TNF-α	hyperplasia of Kupffer cells, oxidative stress of the hepatocytes
Cell death	Lead acetate, 1 mg/ml	1 week	Female mice	Overexpression of apoptotic markers like Bax, Caspase 8, Caspase 3	apoptosis, oxidative stress

**Table 6:** Biosorption by different microbes. The table is optimized after [162].

Microbial Biosorbent	pH	Temperature (°C)	Time (h)	Initial Metal Ion Concentration (mg/L)	Sorption Capacity (mg/g)
*Enterobacter cloacae*	8	40	72	400	172
*Pseudomonas aeruginosa*	7.5	40	24	50	40
*Micrococcus luteus*	-	27	48	272	1.965
*Aspergillus niger*	4.5	30	72	100	34.4
*Aspergillus fumigatus*	4	30	48	100	35
*Saccharomyces cerevisiae*	8	60	6	98.25	80
*Phanerochaete chrysosporium*	6	20	1	100	88.16
*Botrytis cinerea*	4	25	1.5	350	107.1

## Data Availability

No new data were created or analyzed in this study.

## Abbreviations

The following abbreviations are used in this manuscript:

OSHA	Occupational Safety and Health Administration
RND	Resistance-Nodulation-Division (family)
MICP	Microbially Induced Carbonate Precipitation
ZIP	ZRT-IRT-like Protein (family)
NRAMP	Natural Resistance-Associated Macrophage Protein
ROS	Reactive Oxygen Species
FAO	Food and Agriculture Organization (of the United Nations)
GSH/MRP	Glutathione/Multidrug Resistance-associated Protein
